# Influence of cognitive functions on central auditory processing tests in university students

**DOI:** 10.1590/2317-1782/e20250036en

**Published:** 2026-03-30

**Authors:** Thaís Andreza Oliveira Barbosa, Sebastian Ariel Jimenez Cortes, Luciana Cássia de Jesus, Luciana Macedo de Resende, Luciana Mendonça Alves

**Affiliations:** 1 Departamento de Fonoaudiologia, Universidade Federal de Minas Gerais – UFMG - Belo Horizonte (MG), Brasil.

**Keywords:** Auditory Perception, Auditory Processing, Cognition, Adult, Students, Neuropsychological Tests

## Abstract

**Purpose:**

To analyze the influences of cognitive functions on central auditory processing (CAP) tests in university students, through a brief neuropsychological assessment.

**Methods:**

Observational, analytical, cross-sectional study with 95 undergraduate and postgraduate students aged 18 to 39 years. They underwent otoscopy, basic audiological evaluation, behavioral assessment of auditory processing using the Speech-in-Noise, Pitch Pattern Sequence (PPS), Duration Pattern Sequence (DPS), Random Gap Detection Test (RGDT), Masking Level Difference (MLD), Dichotic Digits Test (DDT), and neuropsychological assessment using the Brief Neuropsychological Assessment Instrument (NEUPSILIN). The chi-square test analyzed the association of variables, followed by a logistic regression model to estimate the odds ratio between variables with statistical significance.

**Results:**

There were statistically significant associations between the RGDT result, attention, and verbal fluency, and between the DDT and working memory. Moreover, abnormal verbal fluency increases the odds of abnormal RGDT results by 4.7 times, and abnormal working memory and problem-solving increase the odds of abnormal DDT results by 8 times and 6.6 times, respectively.

**Conclusion:**

Working memory, attention, cognitive planning, processing speed, and cognitive flexibility particularly influenced the temporal resolution and dichotic listening tests. Further research may expand this reflection on the interpretative analysis of CAPD tests and diagnosis.

## INTRODUCTION

Central auditory processing (CAP) can be defined as the way the central nervous system (CNS) processes auditory information, supported by its neurobiological activities. It encompasses the skills of sound localization and lateralization, auditory discrimination, auditory pattern recognition, temporal patterns of hearing, and auditory performance with concurrent or degraded acoustic signals. Thus, CAP disorder (CAPD) refers to difficulties in processing auditory information in the CNS, characterized by a deficit in the performance of one or more of the skills mentioned above^(1)^.

It is known that adequate auditory development is an essential factor for the development of oral and written language. Several factors can influence auditory performance, such as extrinsic factors (socio-economic and environmental conditions, chemicals and drugs, exposure to music) and intrinsic factors (language alterations and neurological alterations). Extrinsic factors can modify the person’s auditory experiences, influencing the development of their system, while intrinsic factors can generate difficulties in functions that are necessary for good auditory development^(2)^.

CAPD can occur in isolation or coexist with other disorders, such as attention-deficit/hyperactivity disorder (ADHD), language disorder, and learning disorders^(1)^. However, making a differential diagnosis of such comorbidities requires caution because CAPD is not due to higher mental and language dysfunctions, although these disorders can coexist. Certain contexts, such as acoustically challenging environments, recruit higher cognitive functions along with auditory skills^(2,3)^, whereas CAPD occurs due to a dysfunction in the neural processing of auditory information, manifesting various symptoms, such as difficulty understanding speech in noise, following auditory commands, difficulty maintaining attention, and learning difficulties. Such manifestations make differential diagnosis difficult between this and other disorders that may present similar symptoms^(1,2)^.

Although these manifestations are very common in school-age children and adolescents, CAPD can also occur in adults as a disorder that has persisted since childhood or is associated with specific events, such as strokes, head injuries, and exposure to neurotoxins^(3)^. Thus, CAPD-related symptoms and difficulties can also impair adults in academic situations.

A Brazilian study aimed at validating the Central Auditory Processing Skill Self-Perception Scale (CAPSSPS) in adults demonstrated that most of the volunteers’ complaints were related to academic, non-auditory factors. However, after the CAP behavioral assessments for temporal resolution and auditory closure skills, the scale showed that the score of the complaints in the self-perception questionnaire was associated with the auditory alterations presented in the CAP tests^(4)^.

It is important to emphasize that the results of CAP tests can be influenced by factors other than hearing, such as the method of application, the motivational factor of the individual being evaluated, and cognitive functions^(1)^. The latter are abilities that process cortical information and involve complex mental processes, such as memory, learning, thinking, attention, language, and executive functions^(3,5)^. Executive functions integrate, coordinate, regulate, and optimize cognitive processes to achieve a goal, such as maintaining attention or controlling behavior. Several theoretical models address which cognitive components are part of executive functions. However, three main components (working memory, inhibitory control, and cognitive flexibility) serve as the basis for maintaining attention and controlling behavior^(6)^.

We use working memory when we need to modify, store, and recall information to perform different tasks^(7)^. Thus, it both stores and manipulates information^(8)^. Inhibitory control allows the individual to inhibit an automatic response in order to perform behavioral or cognitive control and achieve a goal^(6)^. Lastly, cognitive flexibility is the ability to switch between different concepts or resolutions of cognitive tasks^(6)^.

As mentioned earlier, auditory processing assessment can be influenced by cognitive factors. Different studies seek to analyze the cognitive functions that may be related to performance on CAP tests by both children^(2,9,10,11)^ and adults^(3,7)^. A 2023 systematic review analyzed the results of 126 articles that described the relationship between cognitive abilities and auditory processing in adults. The study included cognitive function tasks of working memory, processing speed, executive/inhibitory functions, fluid intelligence, visual perception, and multi-domain cognitive functions^(3)^. Although some of these fall within the concept of executive functions, the review showed that some studies analyzed each cognitive task separately, while others did not specify the executive function evaluated. Furthermore, the authors found that there is no consensus in the literature on which cognitive processes have the greatest influence on auditory processing, since the included studies found significant and non-significant results, although they analyzed the same function.

However, despite the variability of cognitive functions studied in relation to CAP and the heterogeneity of results, some functions stand out in the literature investigating the relationship between cognition and CAP, such as working memory, attention, processing speed, and inhibitory control^(3,7,9-12)^. Because working memory allows the manipulation of received information^(8)^, it is one of the most studied cognitive functions regarding its association with auditory processing. A 2023 review study observed that 58 of the 126 articles it included researched working memory and its association with CAP, highlighting it as one of the most cited cognitive functions regarding its relationship with auditory processing^(3)^.

Similarly, the literature cites attention as an important factor for performing CAP tests^(9-11)^. Some studies suggest that dichotic listening may be related to attention, especially when using auditory attentional and divided attention tests^(9,11)^. Gyldenkærne et al.^(10)^, in a study with 101 children, also suggested that sustained attention tests, including visual modality tests, correlate with dichotic listening, assessed by the Dichotic Digits Test (DDT), and with temporal ordering, assessed by the Frequency Pattern Test (FPT). Despite the different findings regarding the attentional type, the studies corroborate the idea that attention, in its different forms, plays an important role in performing auditory processing tests and, therefore, should be considered when making the diagnosis of CAPD and the differential diagnosis of attentional disorders^(9-11)^.

Furthermore, processing speed, which refers to the speed at which information is processed at the cortical level to complete a task, can involve tasks related to hearing, such as listening in noisy environments or with degraded auditory information, and tasks related to language, such as lexical processing speed. Thus, this cognitive function is studied especially in relation to auditory skills that interfere with speech in noise or with complex information, being also cited as an important factor for the ability to process auditory information during aging^(3,7)^.

Furthermore, inhibitory functions, or inhibitory control, which are part of executive functions, are also instruments of interest in some studies in the area because they allow the individual to maintain focus on a particular activity, which can be recruited during listening in acoustically unfavorable environments^(3,7)^.

Finally, according to Davidson and Souza^(3)^, auditory closure and temporal processing skills are the most significantly associated with cognitive domains in the literature.

Therefore, professionals performing behavioral assessments of auditory processing must know how different cognitive aspects can influence CAP test results. It is understood that, along with peripheral hearing, cognition and auditory processing skills allow interpretation of and response to different acoustic signals, which can influence learning and communicative performance^(3,12,13)^. A Brazilian study evaluated adults’ auditory and academic complaints and highlighted a higher prevalence of complaints related to executive functions (e.g., concentration, planning, and memory) among those who reported academic complaints. Such difficulties may be related to auditory factors, since these individuals were more likely to present auditory difficulties than those who did not present academic complaints^(13)^.

Thus, considering the academic, linguistic, and communicative demands of university settings, which can be influenced by CAP, such studies must address the university population. It is also necessary to consider that memory, attention, executive functions, and other cognitive aspects can influence the results of auditory assessments^(3,7,9-12)^. Therefore, this study aimed to analyze the influence of cognitive functions on CAP behavioral test results through a brief neuropsychological assessment battery.

## METHODS

### Ethical aspects and study type and location

The study was approved by the Research Ethics Committee (COEP) of the educational institution, under approval number 6,470,575. An informed consent form was obtained in accordance with CEP resolution 466/2012 for human research.

This cross-sectional, analytical, observational study was carried out at a public higher education institution, with a non-probabilistic sample of undergraduate and postgraduate students. The participants were volunteers who signed an informed consent form.

### Sample selection and inclusion and exclusion criteria

The study was divided into two stages, the first conducted online via a form sent by email, and the second in person. The online stage served to invite interested students to participate in the study, collect their sociodemographic data, and obtain their informed consent. Then, volunteers were selected by convenience to participate in the in-person assessment, which aimed to evaluate peripheral hearing, CAP, and cognitive aspects. Details of the stages are described in Procedures and Instruments below.

The inclusion criteria were Brazilian undergraduate or postgraduate university students of any race or sex, aged 18 to 39 years, with informed consent. This information was collected from the electronic form. The study excluded volunteers whose in-person evaluation with the four-tone average at 500, 1000, 2000, and 4000 Hz classified them with hearing loss, according to the criteria of the World Health Organization^(14)^, and/or had types As, Ad, B, or C tympanograms^(15,16)^.

### Procedures and instruments

For the first stage of the research, a form was sent via institutional email to undergraduate and postgraduate students at the institution where the research was conducted. The form was used to collect sociodemographic data and contained the study objectives and an informed consent form, asking students to indicate whether they were interested in participating in the research. It also contained a field for filling in data regarding name, age, education, nationality, telephone number, and email address for contact.

Next, the data collected in the form were tabulated in an Excel spreadsheet, from which the volunteers who met the inclusion criteria for the study were analyzed. Based on this result, volunteers were selected by convenience to attend the in-person evaluation at the speech-language-hearing laboratory of the institution where the research was conducted. The authors selected them randomly, based on the numbers assigned to the volunteers at the time of data entry into the Excel spreadsheet. However, the number of volunteers selected was non-probabilistic and was not defined at the beginning of data collection, since the evaluations took place according to the authors' availability for data collection. Thus, volunteers were called for in-person evaluations, which took place over approximately 1 year, and the authors conducted one to two evaluations per week, on average. At the end of the stipulated period, the evaluations were finalized with the number of volunteers obtained.

The in-person stage included basic audiological assessment, auditory processing tests, and neuropsychological assessment.

The basic audiological assessment aimed to evaluate the volunteers’ peripheral hearing. Otoscopy was performed to analyze the integrity of the external auditory canal and tympanic membrane, bilaterally, assessing whether there was any impediment to performing the examination. Then, impedance audiometry and pure-tone audiometry (PTA) were performed. Impedance audiometry was performed with the AT235h device, Interacoustics brand, to research tympanometry and contralateral stapedial reflexes. Tympanometry aimed to evaluate the functioning and integrity of the middle ear, bilaterally, with the 226-Hz test tone. Participants with type As, Ad, C, or B tympanograms, according to Jerger^(15)^ and Jerger et al.^(16)^, were excluded, since these results indicate possible middle ear alteration. PTA was performed using the Inventis Piano device to define the hearing threshold at 250, 500, 1000, 2000, 3000, 4000, 6000, and 8000 Hz via air conduction and at 500, 1000, 2000, 3000, and 4000 Hz via bone conduction, when necessary. Based on the four-tone average of the thresholds at 500, 1000, 2000, and 4000 Hz, their audiometry was classified according to the World Health Organization^(14)^, and the presence of any degree of hearing loss was considered an exclusion criterion for the study.

Immediately afterwards, a CAP behavioral assessment was carried out, evaluating each test according to its normality pattern^(17-21)^. The results were described in binary categories of "normal" or "abnormal".

In the category of low-redundancy monaural tests, the Speech in Noise test^(17)^ was used to assess auditory closure ability. It was performed at 40 dB SL (dB sensation level) above the three-tone average, with an ipsilateral signal-to-noise ratio (SNR) of -5 dB. Results were considered normal when there were 70% or more correct answers, with less than 20% difference between the results with and without noise^(17)^.

In the temporal processing tests category, the Pitch Pattern Sequence (PPS)^(18)^ and the Duration Pattern Sequence (DPS)^(18)^ were used to assess temporal ordering, and the Random Gap Detection Test (RGDT)^(19)^ was used to assess temporal resolution. All three were presented at 50 dB above the three-tone average. Temporal ordering tests were performed monotically, with a naming-type response. Normality was in accordance with the standards of the version used^(18)^, with 88% or more correct answers for the PPS and 82% or more for the DPS, not considering inversion as an error. Thus, results with a value equal to or above these normality values were considered normal^(18)^. The RGDT test was performed binaurally, and normality was defined according to the detection threshold found, which should be equal to or less than 20 ms, according to Keith's standards^(19)^.

In the binaural interaction testing category, the Masking Level Differences (MLD) test was used^(20)^. The test was performed binaurally at 50 dB above the three-tone average. Normality was defined as a minimum of 10 ms, according to the standards of Wilson et al.^(20)^. Thus, volunteers whose results were equal to or greater than 10 ms were described as normal.

In the dichotic listening test category, the DDT^(21)^ was used to assess auditory figure-ground ability. The test was performed in the binaural integration task. The normality criterion used was 95% or more correct answers^(21)^, so normal results should be equal to or above this value.

Finally, the study used the Brief Neuropsychological Assessment Instrument (NEUPSILIN)^(22)^, which consists of a test with several cognitive tasks that are easy to solve and quick to administer (between 30 and 40 minutes). The tasks performed assessed temporospatial orientation, attention, memory, arithmetic skills, and executive functions (problem solving and verbal fluency). The choice of tasks was based on findings from studies in the literature that investigate the relationship between these skills and their influence on auditory processing^(3,5,9)^. Quantitative analysis was performed, considering the Z-scores of the functions, except for the memory function, where the Z-score of the working memory task was also analyzed. Each function was evaluated according to the volunteer’s raw score, which was used to calculate the Z-score, considering the group's average value in the test and the standard deviation of their normative group – i.e., adults aged 18 to 39 with more than 9 years of education, as these groups correspond to the profile of the study volunteers. Thus, their performances were classified into normal, suggestive of a warning sign for deficit, suggestive of deficit, suggestive of moderate to severe deficit, and suggestive of significant deficit^(22)^. The performance classifications were transformed into binary categories to facilitate the analysis of the study data; hence, normal results were those that had a Z-score within the “normal” category, and abnormal results were those that had a Z-score within the categories suggestive of “warning sign for deficit”, “deficit”, “moderate to severe deficit”, and “significant deficit”.

The neuropsychological assessment was carried out after the auditory tests. The research environment was air-conditioned, and care was taken to minimize fatigue from administering the tests.

### Analysis of the results

The analysis of the results was ordered in three parts. First, a descriptive analysis of the sample was performed. Qualitative variables were presented in absolute and relative frequencies, while quantitative variables underwent the Shapiro-Wilk test. From this analysis, the quantitative data were presented in medians and quartiles.

Next, the chi-square test assessed the relationship between auditory processing tests and neuropsychological assessment, in which the significance level of the analyses was 5%. Thus, all results with p-values less than 0.05 were considered statistically significant.

Based on the results of the chi-square test analysis, a logistic regression model was used to evaluate the odds ratio of variables related to cognitive functions on CAP tests. Each model included all independent variables from the neuropsychological test in relation to the CAP tests that had a p-value less than 0.2 in the chi-square test. In other words, the regression model was generated from all variables whose p-values were < 0.2. However, only the variables that reached a p-value < 0.05 in the mathematical model remained in the regression model, making it possible to evaluate their odds ratio. All variables with a p-value less than 0.2 were included because variables with this value can reach p < 0.05 within the regression model when analyzed together with other variables. Likewise, variables with a p-value < 0.05 in isolation in the chi-square test may no longer present such statistical significance when included with other variables in the regression model. The objective of the logistic regression was to evaluate the probability of alterations in the CAP tests based on the neuropsychological test results.

The analyses were performed using IBM SPSS, version 25.

## RESULTS

**Figure 1:**
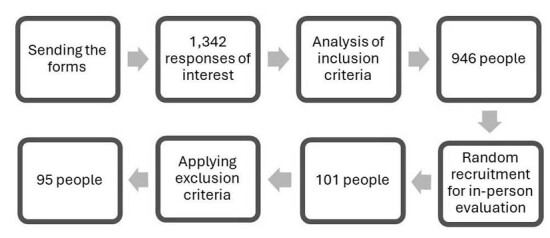
Flowchart of the research stages

After submitting the application form, 1,342 responses were received from students interested in volunteering for the study, of which 946 met the inclusion criteria. Of these, 101 students participated in the in-person assessment. After applying the exclusion criteria, the study obtained and analyzed 95 assessments. Six volunteers were excluded due to hearing loss (one participant) and types B and C tympanograms (five volunteers). The flowchart in Figure 1 shows the number of participants per stage.

Of the 95 research participants, 26 (27.7%) were male, and 69 (72.6%) were female. Also, 55 (57.8%) were undergraduate, and 40 (42.1%) were postgraduate students (master's and doctoral); 60 volunteers (63.2%) came from public high schools, while 35 volunteers (36.8%) came from private high schools. The sample’s median age was 25 years, with the first quartile being 23 years and the third quartile being 30 years.

The auditory processing and NEUPSILIN test results were described in binary categorical variables, described as "normal" or "abnormal." Auditory processing test results outside the reference standard of each test were considered abnormal. Similarly, neuropsychological test results different from "normal" after analysis of the NEUPSILIN Z-scores were considered abnormal, including those described as suggestive of “warning sign for deficit”, “deficit”, “moderate to severe deficit”, and “significant deficit.” Tables 1 and 2 present the assessment results.

**Table 1:** Results of the central auditory processing tests (n = 95)

**Variables**		**n**	**%**
**Speech-in-Noise**	Normal	64	67.4
	Abnormal	31	32.6
**MLD**	Normal	74	77.9
	Abnormal	21	22.1
**PPS**	Normal	60	63.2
	Abnormal	35	36.8
**DPS**	Normal	77	81.1
	Abnormal	18	18.9
**RGDT**	Normal	82	86.3
	Abnormal	13	13.7
**DDT**	Normal	86	90.5
	Abnormal	9	9.5

**Caption:** MLD = Masking Level Difference; PPS = Pitch Pattern Sequence; DPS = Duration Pattern Sequence; RGDT = Random Gap Detection Test; DDT = Dichotic Digits Test; Normal = results considered normal according to the reference standard of each test; Abnormal = results considered abnormal according to the reference standard of each test

**Table 2:** NEUPSILIN results (n = 95)

**Variables**		**n**	**%**
**Spatiotemporal orientation**	Normal	86	90.5
	Abnormal	9	9.5
**Attention**	Normal	93	97.9
	Abnormal	2	2.1
**Memory**	Normal	84	88.4
	Abnormal	11	11.6
**Working memory**	Normal	86	90.5
	Abnormal	9	9.5
**Arithmetic skills**	Normal	76	80.0
	Abnormal	19	20.0
**Language**	Normal	85	89.5
	Abnormal	10	10.5
**Executive functions (problem solving)**	Normal	85	89.5
	Abnormal	10	10.5
**Executive functions (verbal fluency)**	Normal	84	88.4
	Abnormal	11	11.6

**Caption:** Normal = results within the "normal" classification, according to the test's reference standard; Abnormal = results within the categories suggestive of “warning sign for deficit”, “deficit”, “moderate to severe deficit”, and “significant deficit”, according to the test's reference standard

An association analysis was performed between the binary categorical variables of the auditory processing tests and the neuropsychological tests. However, the cognitive functions assessed were not statistically significantly associated with the Speech-in-Noise, PPS, DPS, and MLD tests. RGDT and DDT were statistically significantly associated with some of the neuropsychological variables studied.

In the association analysis between the RGDT and the neuropsychological assessment, two variables had statistically significant results (p-value < 0.05), namely the attention task (p-value = 0.017) and the verbal fluency task (p-value = 0.041). It is important to emphasize that all cases (100%) with a normal RGDT result also had a normal result in the attention task. Table 3 shows the results of the association between the RGDT and the neuropsychological assessment.

**Table 3:** Results of the association between RGDT and neuropsychological assessment

**Variable**	**RGDT**		
	**Normal**	**Abnormal**	**p-value** ^*^
**Spatiotemporal orientation**			
Normal	73 (89%)	13 (100%)	0.353
Abnormal	9 (11%)	0 (0%)	
**Attention**			
Normal	82 (100%)	11 (84.6%)	**0.017**
Abnormal	0 (0%)	2 (15.4%)	
**Memory**			
Normal	74 (91.5%)	10 (76.9%)	0.172
Abnormal	8 (9.8%)	3 (23.1%)	
**Working memory**			
Normal	75 (91.5%)	11 (84.6%)	0.606
Abnormal	7 (8.5%)	2 (15.4%)	
**Arithmetic skills**			
Normal	66 (80.5%)	10 (76.9%)	0.719
Abnormal	16 (19.5%)	3 (23.1%)	
**Language**			
Normal	73 (89%)	12 (92.3%)	0.999
Abnormal	9 (11%)	1 (7.7%)	
**Problem solving**			
Normal	75 (91.5%)	10 (76.9%)	0.136
Abnormal	7 (8.5%)	3 (23.1%)	
**Verbal fluency**			
Normal	75 (91.5%)	9 (69.2%	**0.041**
Abnormal	7 (8.5%)	4 (30.8%)	

* Chi-square test

Caption: RGDT = Random Gap Detection Test

Besides the RGDT, the DDT was also statistically significantly associated with one of the NEUPSILIN variables, namely working memory (p-value = 0.038), as shown in Table 4.

**Table 4:** Results of the association between the Dichotic Digits Test and neuropsychological assessment

**Variables**	**Dichotic Digits Test**		
	**Normal**	**Abnormal**	**p-value** ^*^
**Spatiotemporal orientation**			
Normal	77 (89.5%)	9 (100%)	0.594
Abnormal	9 (10.5%)	0 (0%)	
**Attention**			
Normal	85 (98.8%)	8 (88.9%)	0.181
Abnormal	1 (1.2%)	1 (11.1%)	
**Memory**			
Normal	77 (89.5%)	7 (77.8%)	0.279
Abnormal	9 (10.5%)	2 (22.2%)	
**Working memory**			
Normal	80 (93%)	6 (66.7%)	**0.038**
Abnormal	6 (7%)	3 (33.3%)	
**Arithmetic skills**			
Normal	70 (81.4%)	6 (66.7%)	0.377
Abnormal	16 (18.6%)	3 (33.3%)	
**Language**			
Normal	78 (90.7%)	7 (77.8%)	0.240
Abnormal	8 (9.3%)	2 (22.2%)	
**Problem solving**			
Normal	79 (91.9%)	6 (66.7%)	0.051
Abnormal	7 (8.1%)	3 (33.3%)	
**Verbal fluency**			
Normal	76 (88.4%)	8 (88.9%)	0.999
Abnormal	10 (11.6%)	1 (11.1%)	

* Chi-square test

Next, a logistic regression model was applied to all associations between the CAP tests and neuropsychological variables with a p-value < 0.2 to investigate their relationship. Then, the logistic model evaluated which variables reached a p-value < 0.05 and presented the odds ratio between them – i.e., the probability that those with abnormal neuropsychological test results also had abnormal CAP test results, in comparison with those who did not have abnormal results in these neuropsychological variables. No neuropsychological variable analyzed with the Speech-in-Noise, PPS, DPS, and MLD tests reached a p-value < 0.05 in the logistic regression model; thus, no model was presented for these tests.

Attention, in isolation, was statistically significantly associated with RGDT in the chi-square test. However, when the logistic regression model was applied to the variables with a p-value < 0.2 in relation to RGDT, only verbal fluency remained in the mathematical model with a p-value < 0.05, making it possible to evaluate its odds ratio. Thus, it was demonstrated that abnormal verbal fluency increases the odds of abnormal RGDT by 4.7 times, compared to normal verbal fluency. The classification capacity of the model was 86.3% - i.e., almost all the estimates generated by the regression coincide with the real result of the variable.

Furthermore, a second regression model, this time in relation to DDT, obtained variables with a p-value < 0.05 at the end, making it possible to evaluate their odds ratio. The model demonstrated that abnormal working memory and problem-solving increase the chance of having an abnormal DDT result by almost 8 times and 6.6 times, respectively, compared to normal results in these cognitive functions. These results are shown in Table 5.

**Table 5:** Logistic regression model

	**OR**	**Wald**	**df**	**p-value**	**95% CI for OR**		**Classification Table**
**Response variable: RGDT**							
Constant	0.120	36.125	1	0.000			
Verbal fluency	4.762	4.708	1	0.030	1.163	19.498	86.3%
**Response variable: Dichotic Digits Test**							
Constant	0.051	32.888	1	0.000			
Working memory	7.814	5.469	1	0.019	1.395	43.770	91.6%
Problem solving	6.652	4.762	1	0.029	1.213	36.479	

**Caption:** OR = odds ratio; Classification table: explanatory power of the model

Tables with the results of the association analysis of the other auditory processing tests – Speech-in-Noise, PPS, DPS, and MLD – with the cognitive functions evaluated are available as Supplementary Material. We encourage all readers to consult them to verify the results of each test.

## DISCUSSION

This study aimed to analyze the influence of cognitive functions on CAP test results in university students. This topic is important because different cognitive processes can influence the assessment of auditory processing, and it is necessary to know these processes to assess individuals more accurately^(1,3)^.

In this study, the population consisted of undergraduate and postgraduate university students; most volunteers were women, undergraduate students, and public high school graduates. Several studies in the literature address cognitive aspects and CAP in children^(2,9,10)^; however, university academic students are not yet the focus of research, although the population is also the target of academic and auditory complaints^(4,13)^.

The literature demonstrates a variability of cognitive functions that can influence the assessment of auditory processing^(3)^. Initially, the study results found a statistically significant association between temporal resolution and attention and verbal fluency tasks. However, using the logistic regression model, only verbal fluency remained related to abnormal RGDT test results.

A 2023 systematic literature review demonstrated that temporal ordering was more related, in the included studies, to the cognitive processes of working memory, intelligence tests, and global cognitive measures, although it also pointed to studies that did not obtain this association. Most studies in the literature addressing the relationship between temporal processing and cognitive functions used temporal ordering tests for evaluation^(3,23,24)^. Most other studies that analyzed temporal resolution did not find statistically significant associations between this ability and different cognitive domains, such as attention, memory, and intelligence^(3, 24)^.

In this sense, the present study went against the literature, as it found no associations between the cognitive functions studied and temporal ordering, assessed by the PPS and DPS tests. Nonetheless, most participants with normal results in these tests also had normal results in the attention and working memory tasks. Temporal resolution, assessed by the RGDT, was statistically significantly associated with the attention and verbal fluency tasks. The result, which contrasts with the literature, can be explained by the fact previously stated that most research does not aim to study temporal resolution, besides the small sample size in such studies^(3,24)^.

Although it did not remain in the logistic regression model along with verbal fluency, the statistically significant association between the RGDT and attention in the chi-square test indicates that this may be an important cognitive function for performing the temporal resolution task. In the RGDT, the subject must identify whether they heard one or two tones among a series of stimuli^(19)^. Although the test time is not long, the result indicating that attention plays a crucial role in its performance is plausible, suggesting that this is a necessary function for consistent assessment responses. Thus, attention appears to be a protective factor, positively associated with adequate RGDT performance, and can be considered an important function for performing this test.

In addition to attention, verbal fluency also had statistically significant results and remained in the regression model as a variable with a strong influence on the RGDT. According to the results, there is a greater tendency to abnormal RGDT results (approximately 4.7 times more likely) when the verbal fluency test results are abnormal. The NEUPSILIN indicates verbal fluency as a test of executive functions, which include a series of cognitive processes, such as working memory, inhibitory control, and cognitive flexibility^(6)^. These are important for task management, planning, and monitoring of other cognitive functions, such as sustained attention. The verbal fluency task is one of the main indicators used to assess executive functions, since it involves the ability to organize thought and speed of lexical production and access to the lexicon^(25)^, which suggests that the cognitive processes of planning and speed of thought are involved in the performance of both tests and are important functions in RGDT performance. Thus, difficulty in cognitive planning and speed of thought may increase the chances of abnormal temporal resolution task results. However, this result must be evaluated cautiously, since it is not a frequent finding in the literature.

Among cognitive functions, working memory is one of the most studied factors in relation to its influence on auditory processing test performance^(3)^. In this study, working memory and problem-solving demonstrated influence, specifically, on DDT performance. Dichotic listening refers to the ability to separate or integrate information from different auditory stimuli presented simultaneously to both ears^(1)^. According to the regression model results, abnormal working memory increases the odds of abnormal DDT results by 8 times, compared to when there are no difficulties in working memory. Other studies have already pointed to the importance of this function for dichotic listening, using different tests, whether in the binaural integration or separation task^(9,26)^. During the performance of dichotic listening tests, the evaluator must be aware that working memory is recruited in response to dichotic stimuli, which can influence the person’s performance. Although NEUPSILIN assesses working memory separately from executive functions, it is one of its main components. It enables the manipulation of information^(6)^, including auditory information, as required in dichotic listening tasks^(9,26)^.

In addition to working memory, the regression model indicated that abnormal results in the problem-solving task increase the odds of abnormal DDT results by 6.6 times. This finding suggests that cognitive processes of planning, prioritization, strategy, and cognitive flexibility are also involved in responding to dichotic stimuli. This reiterated the need to consider executive functions when assessing dichotic listening, especially if the assessment aims for the differential diagnosis of CAPD and other disorders, such as ADHD, in which difficulties with executive functions are common^(3,9,11,27,28)^.

Literature also indicates a relationship between auditory closure and some cognitive abilities, especially the executive functions of inhibitory control and working memory^(3,7,12)^. The present study did not find a statistically significant association between these variables. However, this result can be explained by the fact that the research that found such associations used low-redundancy monaural speech tests structured with sentences and with complex or degraded auditory signals^(3,7,12)^. The Speech-in-Noise test consists of repeating monosyllables ipsilaterally with white noise. Thus, the lack of association between the Speech-in-Noise test and the cognitive functions evaluated suggests that the immediate repetition of monosyllables, even in noise, does not recruit working memory and inhibitory control functions in the same way as other tests in the same category.

It is important to emphasize that the study has the weakness of recruiting a sample without a sample size calculation. Thus, 95 undergraduate and postgraduate students were recruited by convenience sampling, with a large percentage of normal results. Future studies need sample size calculation to evaluate the study population representatively. More specific neuropsychological tests should also be used for the cognitive functions that proved to be most relevant in this study during the evaluation of auditory processing, especially attention and executive functions in their subcategories. Moreover, electrophysiological tests associated with cognitive processes, such as cortical potentials, should complement the evaluation^(3,29)^.

Despite these weaknesses, the importance of this study is recognized, since the population evaluated is not the focus of most research currently found in the literature. Furthermore, there is still no consensus regarding the influence of cognitive functions on auditory processing tests^(3)^. Although some cognitive functions were not statistically significantly associated with performance on some CAP tests, this result suggests that the tests used to assess auditory processing are fulfilling their purpose and are reliable. A test-retest study by Frascá and colleagues^(30)^ had already demonstrated that the assessments have a good level of reliability, and the present study reinforces these findings.

Nevertheless, auditory processing assessment must consider cognitive and neuropsychological aspects to analyze individuals more accurately^(1,5)^. According to the study results, attention, working memory, cognitive planning, processing speed, and cognitive flexibility were related to the CAP tests evaluated, suggesting that behavioral assessment of auditory processing can recruit such cognitive functions. Therefore, multidisciplinary assessment, with speech-language-hearing pathologists, neuropsychologists, and other professionals, is extremely important, especially to clarify the results within the context of a differential diagnosis between disorders.

## CONCLUSION

The literature indicates that different cognitive functions can influence CAP tests in adults. The results of this study suggest that attention, working memory, cognitive planning, processing speed, and cognitive flexibility are recruited during CAP assessment. Hence, abnormal verbal fluency increases the odds of abnormal temporal resolution test results, and abnormal working memory and problem-solving increase the odds of abnormal dichotic listening test results. Furthermore, attention appears to be a protective factor for good performance on the temporal resolution test. Thus, it is essential to consider the performance of these functions during CAP assessment for a more accurate diagnosis of CAPD among adults and university students. Further research can expand this reflection on the interpretative analysis of tests and the diagnosis of CAPD in the study population.

## Material Suplementar

Este artigo acompanha material suplementar.

Tabela suplementar 1 Resultado da associação entre Fala no Ruído e a avaliação neuropsicológica

Tabela suplementar 2 Resultado da associação entre MLD e a avaliação neuropsicológica

Tabela suplementar 3 Resultado da associação entre PPS e a avaliação neuropsicológica

Tabela suplementar 4 Resultado da associação entre DPS e a avaliação neuropsicológica

Este material está disponível como parte da versão online do artigo na página https://doi.org/10.1590/2317-1782/e20250036pt

## References

[1] ASHA: American Speech-Language-Hearing Association (2005). (Central) Auditory processing disorders..

[2] Carvalho NG, Novelli CVL, Colella-Santos MF (2015). Factors in childhood and adolescence that may influence the auditory processing: systematic review. Rev CEFAC.

[3] Davidson A, Souza P (2023). Relationships between auditory processing and cognitive abilities in adults: a systematic review. J Speech Lang Hear Res.

[4] Abreu NCB, de Jesus LC, Alves LM, Mancini PC, Labanca L, de Resende LM (2022). Validation of the Central Auditory Processing Skill Self-Perception Scale (CAPSSPS) for adults. Audiol Commun Res.

[5] Prando ML, Pawlowski J, Fachel JMG, Misorelli MIL, Fonseca RP (2010). Relação entre habilidades de processamento auditivo e funções neuropsicológicas em adolescentes. Rev CEFAC.

[6] Fiske A, Holmboe K (2019). Neural substrates of early executive function development. Dev Rev.

[7] Dryden A, Allen HA, Henshaw H, Heinrich A (2017). The association between cognitive performance and speech-in-noise perception for adult listeners: a systematic literature review and meta-analysis. Trends Hear.

[8] Baddeley A (2012). Working memory: theories, models, and controversies. Annu Rev Psychol.

[9] Riccio CA, Cohen MJ, Garrison T, Smith B (2005). Auditory processing measures: correlation with neuropsychological measures of attention, memory, and behavior. Child Neuropsychol.

[10] Gyldenkærne P, Dillon H, Sharma M, Purdy SC (2014). Attend to this: the relationship between auditory processing disorders and attention deficits. J Am Acad Audiol.

[11] Stavrinos G, Iliadou VM, Edwards L, Sirimanna T, Bamiou DE (2018). The relationship between types of attention and auditory processing skills: reconsidering auditory processing disorder diagnosis. Front Psychol.

[12] Burleson AM, Souza PE (2022). Cognitive and linguistic abilities and perceptual restoration of missing speech: evidence from online assessment. Front Psychol.

[13] Pereira BS, Resende LM, Jesus LC, Escarce AG, Alves LM (2024). Auditory and academic skills self-perception in adults. CoDAS.

[14] WHO: World Health Organization (2021). World report on hearing..

[15] Jerger J (1970). Clinical experience with impedance audiometry. Arch Otolaryngol.

[16] Jerger J, Jerger S, Mauldin L (1972). Studies in impedance audiometry. I. Normal and sensorineural ears. Arch Otolaryngol.

[17] Pereira LD, Schochat E (1997). Processamento auditivo central: manual de avaliação. Acta AWHO..

[18] Auditec (1997). Evaluation manual of pitch pattern sequence and duration pattern sequence..

[19] Keith RW (2008). RGDT Random gap detection test..

[20] Wilson RH, Moncrieff D, Townsend E, Pillion AL (2003). Development of a 500-Hz masking-level difference protocol for clinic use. J Am Acad Audiol.

[21] Pereira LD, Schochat E (2011). Testes auditivos comportamentais para avaliação do processamento auditivo central..

[22] Fonseca RP, Salles JF, Parente MAMP (2009). NEUPSILIN: Instrumento de Avaliação Neuropsicológica Breve..

[23] Rabelo MB, Lopes MS, Corona AP, Carvalho JF, Araújo RPC (2020). Cognitive abilities and performance in the temporal ordering tests for elderly people. Audiol Commun Res.

[24] Murphy CFB, Zachi EC, Roque DT, Ventura DSF, Schochat E (2014). Influence of memory, attention, IQ and age on auditory temporal processing tests: preliminary study. CoDAS.

[25] Silveira DC, Passos LMA, Santos PC, Chiappetta ALML (2009). Avaliação da fluência verbal em crianças com transtorno da falta de atenção com hiperatividade: um estudo comparativo. Rev CEFAC.

[26] Penner IK, Schläfli K, Opwis K, Hugdahl K (2009). The role of working memory in dichotic-listening studies of auditory laterality. J Clin Exp Neuropsychol.

[27] Castro CXL, Lima RF (2018). Consequências do transtorno do déficit de atenção e hiperatividade (TDAH) na idade adulta. Revista Psicopedagogia..

[28] Ferrari J, Lopez Estivalet G, Albuquerque Almeida P (2022). Dificuldades de leitura de estudantes universitários com TDAH: um estudo da influência da memória de trabalho na compreensão leitora. Diacrítica..

[29] Schochat E, Musiek FE, Alonso R, Ogata J (2010). Effect of auditory training on the middle latency response in children with (central) auditory processing disorder. Braz J Med Biol Res.

[30] Frascá MFSS, Lobo IFN, Schochat E (2011). Processamento auditivo em teste e reteste: confiabilidade da avaliação. Rev Soc Bras Fonoaudiol.

