# Supplementary material for: Influence of cognitive functions on central auditory processing tests in university students
Source: Codas. 2026 Mar 30;38(2):e20250036. doi: 10.1590/2317-1782/e20250036en (PMC13075839; doi:10.1590/2317-1782/e20250036en)
Supplement: Tabela suplementar 1 [file codas-38-2-e20250036-suppl01.pdf]

SUPPLEMENTARY MATERIAL

Supplementary Table 1 - Results of the association between the Speech-in-Noise test and neuropsychological assessment

Variables	Speech-in-Noise Test		
	**Normal**	**Abnormal**	**p-value***
**Spatiotemporal orientation**			
Normal	60 (93.8%)	26 (83.9%)	0.146
Abnormal	4 (6.2%)	5 (16.1%)	
**Attention**			
Normal	64 (100%)	29 (93.5%)	0.104
Abnormal	0 (0%)	2 (6.5%)	
**Memory**			
Normal	56 (87.5%)	28 (90.3%)	0.999
Abnormal	8 (12.2%)	3 (9.7%)	
**Working memory**			
Normal	57 (89.1%)	29 (93.5%)	0.713
Abnormal	7 (10.9%)	2 (6.5%)	
**Arithmetic skills**			
Normal	52 (81.3%)	24 (77.4%)	0.662
Abnormal	12 (18.8%)	7 (22.6%)	
**Language**			
Normal	56 (87. 5%)	29 (93.5%)	0.490
Abnormal	8 (12.5%)	2 (6.5%)	
**Executive functions (problem solving)**			
Normal	58 (90.6%)	27 (87.1%)	0.724
Abnormal	6 (9.4%)	4 (12.9%)	
**Executive functions (verbal fluency)**			
Normal	57 (89.1%)	24 (77.4%)	0.745
Abnormal	7 (10.9%)	7 (22.6%)	

*Chi-square test

Supplementary Table 2 - Results of the association between MLD and neuropsychological assessment

Variables	MLD test		
	**Normal**	**Abnormal**	**p-value***
**Spatiotemporal orientation**			
Normal	67 (90.5%)	19 (90.5%)	0.999
Abnormal	7 (9.5%)	2 (9.5%)	
**Attention**			
Normal	73 (97.3%)	21 (100%)	0.999
Abnormal	2 (2.7%)	0 (0%)	
**Memory**			
Normal	67 (90.5%)	17 (81%)	0.253
Abnormal	7 (9.5%)	4 (19%)	
**Working memory**			
Normal	68 (91.9%)	18 (85.7%)	0.409
Abnormal	6 (8.1%)	3 (14.3%)	
**Arithmetic skills**			
Normal	60 (81.1%)	16 (76.2%)	0.621
Abnormal	14 (18.9%)	5 (23.8%)	
**Language**			
Normal	67 (90.5%)	18 (85.7%)	0.687
Abnormal	7 (9.5%)	3 (14.3%)	
**Executive functions (problem solving)**			
Normal	68 (91.9%)	17 (81%)	0.220
Abnormal	6 (8.1%)	4 (19%)	
**Executive functions (verbal fluency)**			
Normal	63 (85.1%)	21 (100%)	0.115

Variables	MLD test		
	**Normal**	**Abnormal**	**p-value***
**Spatiotemporal orientation**			
Normal	67 (90.5%)	19 (90.5%)	0.999
Abnormal	7 (9.5%)	2 (9.5%)	
**Attention**			
Normal	73 (97.3%)	21 (100%)	0.999
Abnormal	2 (2.7%)	0 (0%)	
**Memory**			
Normal	67 (90.5%)	17 (81%)	0.253
Abnormal	7 (9.5%)	4 (19%)	
**Working memory**			
Normal	68 (91.9%)	18 (85.7%)	0.409
Abnormal	6 (8.1%)	3 (14.3%)	
**Arithmetic skills**			
Normal	60 (81.1%)	16 (76.2%)	0.621
Abnormal	14 (18.9%)	5 (23.8%)	
**Language**			
Normal	67 (90.5%)	18 (85.7%)	0.687
Abnormal	7 (9.5%)	3 (14.3%)	
**Executive functions (problem solving)**			
Normal	68 (91.9%)	17 (81%)	0.220
Abnormal	6 (8.1%)	4 (19%)	
**Executive functions (verbal fluency)**			
Abnormal	11 (14.9%)	0 (0%)	

*Chi-square test

Caption: MLD = Masking Level Difference

Supplementary Table 3 - Result of the association between PPS and neuropsychological assessment

Variables	PPS Test		
	Normal	Abnormal	p-value*
**Spatiotemporal orientation**			
Normal	55 (91.7%)	31 (88.6%)	0.721
Abnormal	5 (8.3%)	4 (11.4%)	
**Attention**			
Normal	60 (100%)	33 (94.3%)	0.133
Abnormal	0 (0%)	2 (5.7%)	
**Memory**			
Normal	55 (91.7%)	29 (82.9%)	0.196
Abnormal	5 (8.3%)	6 (17.1%)	
**Working memory**			
Normal	57 (95%)	29 (82.9%)	0.071
Abnormal	3 (5%)	6 (17.1%)	
**Arithmetic skills**			
Normal	51 (85%)	25 (71.4%)	0.111
Abnormal	9 (15%)	10 (28.6%)	
**Language**			
Normal	54 (90%)	31 (88.6%)	0.999
Abnormal	6 (10%)	4 (11.4%)	
**Executive functions (problem solving)**			
Normal	53 (88.3%)	32 (91.4%)	0.741
Abnormal	7 (11.7%)	3 (8.6%)	
**Executive functions (verbal fluency)**			
Normal	54 (90%)	30 (85.7%)	0.529

Variables	PPS Test		
	Normal	Abnormal	p-value*
**Spatiotemporal orientation**			
Normal	55 (91.7%)	31 (88.6%)	0.721
Abnormal	5 (8.3%)	4 (11.4%)	
**Attention**			
Normal	60 (100%)	33 (94.3%)	0.133
Abnormal	0 (0%)	2 (5.7%)	
**Memory**			
Normal	55 (91.7%)	29 (82.9%)	0.196
Abnormal	5 (8.3%)	6 (17.1%)	
**Working memory**			
Normal	57 (95%)	29 (82.9%)	0.071
Abnormal	3 (5%)	6 (17.1%)	
**Arithmetic skills**			
Normal	51 (85%)	25 (71.4%)	0.111
Abnormal	9 (15%)	10 (28.6%)	
**Language**			
Normal	54 (90%)	31 (88.6%)	0.999
Abnormal	6 (10%)	4 (11.4%)	
**Executive functions (problem solving)**			
Normal	53 (88.3%)	32 (91.4%)	0.741
Abnormal	7 (11.7%)	3 (8.6%)	
**Executive functions (verbal fluency)**			
Abnormal	6 (10%)	5 (14.3%)	

*Chi-square test

Caption: PPS = Pitch Pattern Sequence

Supplementary Table 4 - Result of the association between DPS and neuropsychological assessment

Variables	DPS Test		
	Normal	Abnormal	p-value*
**Spatiotemporal orientation**			
Normal	70 (90.9%)	16 (88.9%)	0.678
Abnormal	7 (9.1%)	2 (11.1%)	
**Attention**			
Normal	76 (98.7%)	17 (94.4%)	0.345
Abnormal	1 (1.3%)	1 (5.6%)	
**Memory**			
Normal	69 (89.6%)	15 (83.3%)	0.431
Abnormal	8 (10.4%)	3 (16.7%)	
**Working memory**			
Normal	72 (93.5%)	14 (77.8%)	0.063
Abnormal	5 (6.5%)	4 (22.2%)	
**Arithmetic skills**			
Normal	63 (81.8%)	13 (72.2%)	0.359
Abnormal	14 (18.2%)	5 (27.8%)	
**Language**			
Normal	70 (90.9%)	15 (83.3%)	0.394
Abnormal	7 (9.1%)	3 (16.7%)	
**Executive functions (problem solving)**			
Normal	68 (88.3%)	17 (94.4%)	0.681
Abnormal	9 (11.7%)	1 (5.6%)	
**Executive functions (verbal fluency)**			
Normal	70 (90.9%)	14 (77.8%)	0.211

Variables	DPS Test		
	Normal	Abnormal	p-value*
**Spatiotemporal orientation**			
Normal	70 (90.9%)	16 (88.9%)	0.678
Abnormal	7 (9.1%)	2 (11.1%)	
**Attention**			
Normal	76 (98.7%)	17 (94.4%)	0.345
Abnormal	1 (1.3%)	1 (5.6%)	
**Memory**			
Normal	69 (89.6%)	15 (83.3%)	0.431
Abnormal	8 (10.4%)	3 (16.7%)	
**Working memory**			
Normal	72 (93.5%)	14 (77.8%)	0.063
Abnormal	5 (6.5%)	4 (22.2%)	
**Arithmetic skills**			
Normal	63 (81.8%)	13 (72.2%)	0.359
Abnormal	14 (18.2%)	5 (27.8%)	
**Language**			
Normal	70 (90.9%)	15 (83.3%)	0.394
Abnormal	7 (9.1%)	3 (16.7%)	
**Executive functions (problem solving)**			
Normal	68 (88.3%)	17 (94.4%)	0.681
Abnormal	9 (11.7%)	1 (5.6%)	
**Executive functions (verbal fluency)**			
Abnormal	7 (9.1%)	4 (22.2%)	

*Chi-square test

Caption: DPS = Duration Pattern Sequence

MATERIAL SUPLEMENTAR

Tabela suplementar 1 - Resultado da associação entre Fala no Ruído e a avaliação neuropsicológica

Variáveis	Teste Fala no Ruído		
	Normal	Alterado	valor p*
**Orientação temporo-espacial**			
Normal	60 (93,8%)	26 (83,9%)	0,146
Alterado	4 (6,2%)	5 (16,1%)	
**Atenção**			
Normal	64 (100%)	29 (93,5%)	0,104
Alterado	0 (0%)	2 (6,5%)	
**Memória**			
Normal	56 (87,5%)	28 (90,3%)	0,999
Alterado	8 (12,2%)	3 (9,7%)	
**Memória de trabalho**			
Normal	57 (89,1%)	29 (93,5%)	0,713
Alterado	7 (10,9%)	2 (6,5%)	
**Habilidades aritméticas**			
Normal	52 (81,3%)	24 (77,4%)	0,662
Alterado	12 (18,8%)	7 (22,6%)	
**Linguagem**			
Normal	56 (87, 5%)	29 (93,5%)	0,490
Alterado	8 (12,5%)	2 (6,5%)	
**Funções executivas (resolução de problemas)**			
Normal	58 (90,6%)	27 (87,1%)	0,724
Alterado	6 (9,4%)	4 (12,9%)	
**Funções executivas (fluência verbal)**			
Normal	57 (89,1%)	24 (77,4%)	0,745

Variáveis	Teste Fala no Ruído		
	**Normal**	**Alterado**	**valor p***
**Orientação temporo-espacial**			
Normal	60 (93,8%)	26 (83,9%)	0,146
Alterado	4 (6,2%)	5 (16,1%)	
**Atenção**			
Normal	64 (100%)	29 (93,5%)	0,104
Alterado	0 (0%)	2 (6,5%)	
**Memória**			
Normal	56 (87,5%)	28 (90,3%)	0,999
Alterado	8 (12,2%)	3 (9,7%)	
**Memória de trabalho**			
Normal	57 (89,1%)	29 (93,5%)	0,713
Alterado	7 (10,9%)	2 (6,5%)	
**Habilidades aritméticas**			
Normal	52 (81,3%)	24 (77,4%)	0,662
Alterado	12 (18,8%)	7 (22,6%)	
**Linguagem**			
Normal	56 (87, 5%)	29 (93,5%)	0,490
Alterado	8 (12,5%)	2 (6,5%)	
**Funções executivas (resolução de problemas)**			
Normal	58 (90,6%)	27 (87,1%)	0,724
Alterado	6 (9,4%)	4 (12,9%)	
**Funções executivas (fluência verbal)**			
Alterado	7 (10,9%)	7 (22,6%)	

*Teste Qui Quadrado

Tabela suplementar 2 - Resultado da associação entre MLD e a avaliação neuropsicológica

Variáveis	Teste MLD		
	**Normal**	**Alterado**	**valor p***
**Orientação temporo-espacial**			
Normal	67 (90,5%)	19 (90,5%)	0,999
Alterado	7 (9,5%)	2 (9,5%)	
**Atenção**			
Normal	73 (97,3%)	21 (100%)	0,999
Alterado	2 (2,7%)	0 (0%)	
**Memória**			
Normal	67 (90,5%)	17 (81%)	0,253
Alterado	7 (9,5%)	4 (19%)	
**Memória de trabalho**			
Normal	68 (91,9%)	18 (85,7%)	0,409
Alterado	6 (8,1%)	3 (14,3%)	
**Habilidades aritméticas**			
Normal	60 (81,1%)	16 (76,2%)	0,621
Alterado	14 (18,9%)	5 (23,8%)	
**Linguagem**			
Normal	67 (90,5%)	18 (85,7%)	0,687
Alterado	7 (9,5%)	3 (14,3%)	
**Funções executivas (resolução de problemas)**			
Normal	68 (91,9%)	17 (81%)	0,220
Alterado	6 (8,1%)	4 (19%)	
**Funções executivas (fluência verbal)**			
Normal	63 (85,1%)	21 (100%)	0,115

Variáveis	Teste MLD		
	**Normal**	**Alterado**	**valor p***
**Orientação temporo-espacial**			
Normal	67 (90,5%)	19 (90,5%)	0,999
Alterado	7 (9,5%)	2 (9,5%)	
**Atenção**			
Normal	73 (97,3%)	21 (100%)	0,999
Alterado	2 (2,7%)	0 (0%)	
**Memória**			
Normal	67 (90,5%)	17 (81%)	0,253
Alterado	7 (9,5%)	4 (19%)	
**Memória de trabalho**			
Normal	68 (91,9%)	18 (85,7%)	0,409
Alterado	6 (8,1%)	3 (14,3%)	
**Habilidades aritméticas**			
Normal	60 (81,1%)	16 (76,2%)	0,621
Alterado	14 (18,9%)	5 (23,8%)	
**Linguagem**			
Normal	67 (90,5%)	18 (85,7%)	0,687
Alterado	7 (9,5%)	3 (14,3%)	
**Funções executivas (resolução de problemas)**			
Normal	68 (91,9%)	17 (81%)	0,220
Alterado	6 (8,1%)	4 (19%)	
**Funções executivas (fluência verbal)**			
Alterado	11 (14,9%)	0 (0%)	

*Teste Qui Quadrado

Legenda: MLD = Masking Level Difference

Tabela suplementar 3 - Resultado da associação entre PPS e a avaliação neuropsicológica

Variáveis	Teste PPS		
	**Normal**	**Alterado**	**valor p***
**Orientação temporo-espacial**			
Normal	55 (91,7%)	31 (88,6%)	0,721
Alterado	5 (8,3%)	4 (11,4%)	
**Atenção**			
Normal	60 (100%)	33 (94,3%)	0,133
Alterado	0 (0%)	2 (5,7%)	
**Memória**			
Normal	55 (91,7%)	29 (82,9%)	0,196
Alterado	5 (8,3%)	6 (17,1%)	
**Memória de trabalho**			
Normal	57 (95%)	29 (82,9%)	0,071
Alterado	3 (5%)	6 (17,1%)	
**Habilidades aritméticas**			
Normal	51 (85%)	25 (71,4%)	0,111
Alterado	9 (15%)	10 (28,6%)	
**Linguagem**			
Normal	54 (90%)	31 (88,6%)	0,999
Alterado	6 (10%)	4 (11,4%)	
**Funções executivas (resolução de problemas)**			
Normal	53 (88,3%)	32 (91,4%)	0,741
Alterado	7 (11,7%)	3 (8,6%)	
**Funções executivas (fluência verbal)**			
Normal	54 (90%)	30 (85,7%)	0,529

Variáveis	Teste PPS		
	**Normal**	**Alterado**	**valor p***
**Orientação temporo-espacial**			
Normal	55 (91,7%)	31 (88,6%)	0,721
Alterado	5 (8,3%)	4 (11,4%)	
**Atenção**			
Normal	60 (100%)	33 (94,3%)	0,133
Alterado	0 (0%)	2 (5,7%)	
**Memória**			
Normal	55 (91,7%)	29 (82,9%)	0,196
Alterado	5 (8,3%)	6 (17,1%)	
**Memória de trabalho**			
Normal	57 (95%)	29 (82,9%)	0,071
Alterado	3 (5%)	6 (17,1%)	
**Habilidades aritméticas**			
Normal	51 (85%)	25 (71,4%)	0,111
Alterado	9 (15%)	10 (28,6%)	
**Linguagem**			
Normal	54 (90%)	31 (88,6%)	0,999
Alterado	6 (10%)	4 (11,4%)	
**Funções executivas (resolução de problemas)**			
Normal	53 (88,3%)	32 (91,4%)	0,741
Alterado	7 (11,7%)	3 (8,6%)	
**Funções executivas (fluência verbal)**			
Alterado	6 (10%)	5 (14,3%)	

*Teste Qui Quadrado

Legenda: PPS = Pitch Pattern Sequence

Tabela suplementar 4 - Resultado da associação entre DPS e a avaliação neuropsicológica

Variáveis	Teste DPS		
	Normal	Alterado	valor p*
**Orientação temporo-espacial**			
Normal	70 (90,9%)	16 (88,9%)	0,678
Alterado	7 (9,1%)	2 (11,1%)	
**Atenção**			
Normal	76 (98,7%)	17 (94,4%)	0,345
Alterado	1 (1,3%)	1 (5,6%)	
**Memória**			
Normal	69 (89,6%)	15 (83,3%)	0,431
Alterado	8 (10,4%)	3 (16,7%)	
**Memória de trabalho**			
Normal	72 (93,5%)	14 (77,8%)	0,063
Alterado	5 (6,5%)	4 (22,2%)	
**Habilidades aritméticas**			
Normal	63 (81,8%)	13 (72,2%)	0,359
Alterado	14 (18,2%)	5 (27,8%)	
**Linguagem**			
Normal	70 (90,9%)	15 (83,3%)	0,394
Alterado	7 (9,1%)	3 (16,7%)	
**Funções executivas (resolução de problemas)**			
Normal	68 (88,3%)	17 (94,4%)	0,681
Alterado	9 (11,7%)	1 (5,6%)	
**Funções executivas (fluência verbal)**			
Normal	70 (90,9%)	14 (77,8%)	0,211

Variáveis	Teste DPS		
	Normal	Alterado	valor p*
**Orientação temporo-espacial**			
Normal	70 (90,9%)	16 (88,9%)	0,678
Alterado	7 (9,1%)	2 (11,1%)	
**Atenção**			
Normal	76 (98,7%)	17 (94,4%)	0,345
Alterado	1 (1,3%)	1 (5,6%)	
**Memória**			
Normal	69 (89,6%)	15 (83,3%)	0,431
Alterado	8 (10,4%)	3 (16,7%)	
**Memória de trabalho**			
Normal	72 (93,5%)	14 (77,8%)	0,063
Alterado	5 (6,5%)	4 (22,2%)	
**Habilidades aritméticas**			
Normal	63 (81,8%)	13 (72,2%)	0,359
Alterado	14 (18,2%)	5 (27,8%)	
**Linguagem**			
Normal	70 (90,9%)	15 (83,3%)	0,394
Alterado	7 (9,1%)	3 (16,7%)	
**Funções executivas (resolução de problemas)**			
Normal	68 (88,3%)	17 (94,4%)	0,681
Alterado	9 (11,7%)	1 (5,6%)	
**Funções executivas (fluência verbal)**			
Alterado	7 (9,1%)	4 (22,2%)	

*Teste Qui Quadrado

Legenda: DPS = Duration Pattern Sequence
