# Supplementary material for: Influence of cognitive functions on central auditory processing tests in university students
Source: Codas. 2026 Mar 30;38(2):e20250036. doi: 10.1590/2317-1782/e20250036en (PMC13075839; doi:10.1590/2317-1782/e20250036en)
Supplement: Translation

# Translation: Influência das funções cognitivas nos testes de processamento auditivo central em universitários

**DOI:** 10.1590/2317-1782/e20250036pt

**Authors:** Thaís Andreza Oliveira Barbosa, Sebastian Ariel Jimenez Cortes, Luciana Cássia de Jesus, Luciana Macedo de Resende, Luciana Mendonça Alves

**Affiliations:** 1 Departamento de Fonoaudiologia, Universidade Federal de Minas Gerais – UFMG - Belo Horizonte (MG), Brasil.

**Keywords:** Percepção Auditiva, Processamento Auditivo, Cognição, Adulto, Estudantes, Testes Neuropsicológicos

## Abstract

**Objetivo:**

analisar, por meio de uma avaliação neuropsicológica breve, as influências das funções cognitivas nos testes de Processamento Auditivo Central (PAC) em estudantes universitários.

**Método:**

Estudo observacional, analítico e transversal, composto por 95 estudantes de graduação e pós-graduação entre 18 e 39 anos. Foi realizada meatoscopia, avaliação audiológica básica, avaliação comportamental do processamento auditivo, pelos testes Fala no Ruído (FR), Pitch Pattern Sequence (PPS), Duration Pattern Sequence (DPS), Random Gap Detection Test (RGDT), Masking Level Difference (MLD) e Teste Dicótico de Dígitos (TDD), e avaliação neuropsicológica, com o Instrumento de Avaliação Neuropsicológica Breve - NEUPSILIN. O teste Qui Quadrado analisou a associação das variáveis, seguido do modelo de regressão logística para estimar a razão de probabilidade entre as variáveis com significância estatística.

**Resultados:**

Houve associação estatisticamente significativa entre o resultado do RGDT, atenção e fluência verbal, bem como entre o TDD e a memória de trabalho. Também observou-se que a alteração na fluência verbal aumenta 4,7 vezes a chance da alteração no RGDT e que a alteração na memória de trabalho e na resolução de problemas aumentam em 8 vezes e em 6,6 vezes, respectivamente, a chance de alteração no TDD.

**Conclusão:**

Memória de trabalho, atenção, planejamento cognitivo, velocidade de processamento e flexibilidade cognitiva influenciaram particularmente os testes de resolução temporal e escuta dicótica. Novas pesquisas podem ampliar esta reflexão sobre a análise interpretativa dos testes e o diagnóstico de TPAC.

## INTRODUÇÃO

O Processamento Auditivo Central (PAC) pode ser definido como a maneira com que o Sistema Nervoso Central (SNC) processa informações auditivas, sustentado pelas atividades neurobiológicas desse sistema. Ele abrange as habilidades de localização e lateralização sonora, discriminação auditiva, reconhecimento de padrões auditivos, padrões temporais da audição e desempenho auditivo com sinais acústicos concorrentes ou degradados. Assim, o Transtorno do Processamento Auditivo Central (TPAC) se refere a dificuldades no processamento das informações auditivas no SNC, caracterizado pelo déficit no desempenho de uma ou mais das habilidades citadas anteriormente^(1)^.

Sabe-se que o desenvolvimento auditivo adequado é um fator essencial para o desenvolvimento da linguagem oral e escrita. Ademais, diversos fatores podem influenciar o desempenho auditivo de um indivíduo, como fatores extrínsecos (condições sócio-econômicas e ambientais, químicos e fármacos, exposição à música) e fatores intrínsecos (alterações de linguagem e alterações neurológicas). Isso ocorre, pois fatores extrínsecos podem modificar as experiências auditivas de um indivíduo, influenciando o desenvolvimento do seu sistema, enquanto fatores intrínsecos podem gerar dificuldades em funções que são necessárias para um bom desenvolvimento auditivo^(2)^.

O TPAC pode ocorrer de forma isolada ou pode coexistir com outros distúrbios, como o TDAH (Transtorno do Déficit de Atenção e Hiperatividade), Transtorno da Linguagem ou Transtornos de Aprendizagem^(1)^, entretanto, há de se ter cautela ao realizar o diagnóstico diferencial de tais comorbidades. Isso porque, apesar da possibilidade desses transtornos coexistirem entre si, a causa do TPAC não é devido à existência de disfunções mentais superiores e de linguagem. Apesar de determinados contextos, como ambientes acusticamente desafiadores, recrutarem funções cognitivas superiores junto às habilidades auditivas^(2,3)^, o TPAC ocorre devido a uma disfunção no processamento neural de informações auditivas, o que faz com que diversos sintomas sejam manifestados, como dificuldade para compreender a fala em locais ruidosos, em seguir comandos auditivos, dificuldade em manter a atenção e na aprendizagem. Tais manifestações dificultam o diagnóstico diferencial entre esse e outros transtornos que podem apresentar sintomas semelhantes^(1,2)^.

Embora essas manifestações sejam muito comuns em crianças e adolescentes em idade escolar, o TPAC também pode ocorrer na população adulta, tanto como um transtorno que persistiu desde a infância, quanto associado a eventos específicos, tais como acidentes vasculares cerebrais, traumatismos cranianos e exposição a neurotoxinas^(3)^. Sendo assim, os sintomas e dificuldades relacionadas ao TPAC podem prejudicar, também, a população adulta em situações acadêmicas.

Um estudo brasileiro com intuito de validar a Escala de Autopercepção de Habilidades do Processamento Auditivo Central (EAPAC) em adultos demonstrou que a maior parte das queixas apresentadas pelos voluntários estava relacionada a fatores acadêmicos, não auditivos. Entretanto, após as avaliações comportamentais do PAC para as habilidades de resolução temporal e fechamento auditivo, a escala conseguiu apresentar associação entre a pontuação das queixas apresentadas no questionário de autopercepção com as alterações auditivas apresentadas nos testes de Processamento Auditivo Central^(4)^.

É importante salientar que os resultados dos testes de PAC podem sofrer influência de outros fatores além da audição, como modo de aplicação, fator motivacional do indivíduo avaliado e de funções cognitivas^(1)^. As funções cognitivas se referem a habilidades que permitem o processamento de informações corticais e envolvem processos mentais complexos, como memória, aprendizado, pensamento, atenção, linguagem e funções executivas^(3,5)^. As funções executivas (FE) são responsáveis por integrar, coordenar, regular e otimizar processos cognitivos, a fim de se alcançar um objetivo, como a manutenção da atenção ou controle do comportamento. Existem diversos modelos teóricos que abordam quais componentes cognitivos fazem parte das funções executivas, porém, há três componentes principais que servem de base para haja manutenção da atenção e controle do comportamento, os quais são descritos como memória de trabalho, controle inibitório e flexibilidade cognitiva^(6)^.

A memória de trabalho (memória operacional) é utilizada quando precisamos modificar, armazenar e relembrar informações, a fim de que possamos desempenhar diferentes tarefas^(7)^. Assim, ela não consiste somente no armazenamento de informações, mas também na sua manipulação^(8)^. Já o controle inibitório permite ao indivíduo inibir uma resposta automática, para que ele possa realizar um controle comportamental ou cognitivo, em ordem de se alcançar um objetivo^(6)^. Por fim, a flexibilidade cognitiva se refere à capacidade de alternar entre diferentes conceitos ou resolução de tarefas cognitivas^(6)^.

Como mencionado anteriormente, a avaliação do processamento auditivo pode sofrer influência de fatores cognitivos. Diferentes estudos procuram analisar as funções cognitivas que podem estar relacionadas ao desempenho nos testes de PAC, tanto na população infantil^(2,9,10,11)^, quanto na população adulta^(3,7)^. Uma revisão sistemática de 2023 analisou o resultado de 126 artigos que procuravam descrever a relação entre habilidades cognitivas e processamento auditivo na população adulta. Em relação às funções cognitivas estudadas, foram incluídas, no estudo, as tarefas de memória de trabalho, velocidade de processamento, funções executivas/inibitórias, inteligência fluida, percepção visual e multidomínios cognitivos^(3)^. Nota-se, a partir desta revisão, que, apesar de algumas dessas funções se encontrarem dentro do conceito funções executivas, alguns estudos analisaram cada tarefa cognitiva separadamente, enquanto outros não especificaram a função executiva avaliada. Além disso, os autores constataram que não há um consenso na literatura de quais processos cognitivos mais apresentam influência no processamento auditivo, uma vez que, embora analisassem uma mesma função, verificou-se resultados significativos e não significativos entre as pesquisas incluídas.

Entretanto, apesar da variabilidade de funções cognitivas estudadas em relação ao PAC e da heterogeneidade de resultados, algumas funções se destacam na literatura que investiga a relação entre a cognição e o PAC, como a memória de trabalho, a atenção, a velocidade de processamento e o controle inibitório^(3,7,9-12)^. Devido ao fato de a memória de trabalho permitir a manipulação de informações recebidas^(8)^, ela é uma das funções cognitivas mais estudadas quanto à sua associação com o processamento auditivo. Um estudo de revisão realizado em 2023 observou que, dos 126 artigos incluídos no estudo, 58 englobavam a memória de trabalho na pesquisa de associação com o PAC, evidenciando-a como uma das funções cognitivas mais citadas a respeito de sua relação com o processamento auditivo^(3)^.

Da mesma forma, a atenção é citada na literatura como um fator importante para a realização dos testes de Processamento Auditivo Central^(9-11)^. Alguns estudos sugerem que a escuta dicótica pode estar relacionada à atenção, especialmente quando são utilizados testes atencionais auditivos e de atenção dividida^(9,11)^. Gyldenkærne et al.^(10)^ em uma pesquisa com 101 crianças, também sugeriram que os testes de atenção sustentada, incluindo testes de modalidade visual, apresentam correlação com a escuta dicótica, avaliada pelo Teste Dicótico de Dígitos (TDD), e com a ordenação temporal, avaliada pelo Teste de Padrão de Frequência (TPF). Apesar dos diferentes achados em relação ao tipo atencional, os estudos corroboram com a ideia de que a atenção, em suas diferentes formas, apresenta um papel importante na realização dos testes de processamento auditivo e, portanto, deve ser considerada ao se realizar o diagnóstico de TPAC e o diagnóstico diferencial de transtornos atencionais^(9-11)^.

Além disso, a velocidade de processamento, que se refere à rapidez com que as informações são processadas a nível cortical, a fim de se completar uma tarefa, pode envolver tanto tarefas relacionadas à audição, como a escuta em ambientes ruidosos ou com informações auditivas degradadas, quanto tarefas relacionadas à linguagem, como a velocidade de processamento lexical. Por isso, é uma função cognitiva estudada, especialmente, em relação às habilidades auditivas que interferem na fala no ruído ou com informações complexas, além de ser citada como um fator importante para a capacidade de processar informações auditivas durante o envelhecimento^(3,7)^.

Ademais, as funções inibitórias, ou controle inibitório, as quais fazem parte das funções executivas, também são instrumentos de interesse de alguns estudos na área, devido ao fato de permitirem ao indivíduo que ele mantenha o foco em uma determinada atividade, o que pode ser recrutado durante a escuta em ambientes acusticamente desfavoráveis^(3,7)^.

Por fim, de acordo com Davidson e Souza^(3)^, as habilidades de fechamento auditivo e processamento temporal são as que mais apresentam associação significativa com domínios cognitivos na literatura.

Dessa forma, é necessário que, durante a avaliação comportamental do processamento auditivo, o profissional tenha conhecimento de como diferentes aspectos cognitivos podem influenciar no resultado dos testes de PAC aplicados. Entende-se que, junto da audição periférica, a cognição e as habilidades de processamento auditivo permitem a interpretação e resposta a diferentes sinais acústicos, o que pode influenciar na aprendizagem e no desempenho comunicativo^(3,12,13)^. Um estudo brasileiro que avaliou queixas auditivas e acadêmicas de adultos destacou que, dentre a população que relatava queixas acadêmicas, houve prevalência maior de queixas relacionadas às funções executivas, como de concentração, planejamento e memória. Tais dificuldades podem estar relacionadas a fatores auditivos, uma vez que esses indivíduos tiveram maior tendência a apresentar dificuldades auditivas, em relação aos que não apresentavam queixas acadêmicas^(13)^.

Diante do exposto, considerando as demandas acadêmicas, linguísticas e comunicativas geradas pelo ambiente universitário e tendo em vista que esses fatores podem ser influenciados pelo Processamento Auditivo Central, é importante que a população universitária seja alvo de estudos nessa área. Da mesma forma, é necessário ter em vista que memória, atenção, funções executivas e outros aspectos cognitivos podem influenciar no resultado das avaliações auditivas^(3,7,9-12)^. Portanto, o presente estudo teve como objetivo analisar, por meio de uma bateria de avaliação neuropsicológica breve, a influência das funções cognitivas nos resultados obtidos nos testes comportamentais do Processamento Auditivo Central.

## MÉTODO

### Aspectos éticos, tipo e local do estudo

O estudo foi aprovado pelo Comitê de Ética em Pesquisa (COEP) da instituição de ensino, sob parecer de número 6.470.575. O Termo de Consentimento Livre e Esclarecido (TCLE) foi obtido de acordo com a resolução 466/2012 do CEP para pesquisas com seres humanos.

Trata-se de um estudo observacional analítico transversal, realizado em uma instituição pública de ensino superior, com amostra não probabilística composta de estudantes de nível superior da graduação e pós-graduação. Os participantes foram voluntários, mediante consentimento com o TCLE.

### Seleção da amostra e critérios de inclusão e exclusão

O estudo foi dividido em duas etapas, sendo a primeira realizada em ambiente virtual, via formulário enviado por correio eletrônico, e a segunda, em ambiente presencial. A etapa virtual foi realizada a fim de convidar os alunos interessados a participarem do estudo, coletar os seus dados sociodemográficos e o consentimento com o TCLE. A partir dessa etapa, foram selecionados, por conveniência, os voluntários que participaram da avaliação presencial, que teve como objetivo avaliar a audição periférica, o processamento auditivo central e os aspectos cognitivos. Os detalhes das etapas serão descritos na subseção “Procedimentos e Instrumentos”.

Os critérios de inclusão utilizados foram: ser acadêmico de nível superior, do nível de graduação ou pós-graduação, de qualquer raça e sexo, de nacionalidade brasileira, entre 18 e 39 anos, mediante consentimento com o TCLE. Tais informações foram coletadas a partir do formulário eletrônico. Foram excluídos do estudo os voluntários que, após avaliação presencial, apresentavam classificação de perda auditiva pela média quadritonal das frequências de 500, 1000, 2000 e 4000 Hz, de acordo com os critérios da Organização Mundial de Saúde^(14)^ e/ou apresentar resultados de curva As, Ad, B ou C na imitanciometria^(15,16)^.

### Procedimentos e instrumentos

Para a primeira etapa de pesquisa, foi enviado, via correio eletrônico institucional, um formulário para estudantes de graduação e pós-graduação da instituição de ensino onde a pesquisa foi realizada. O formulário foi utilizado para coletar os dados sociodemográficos e continha os objetivos do estudo, o TCLE e solicitava aos alunos que manifestassem se tinham o interesse em realizar a pesquisa, além de conter um campo para o preenchimento de dados referentes ao nome, idade, escolaridade, nacionalidade, telefone e *e-mail* para contato.

Em seguida, foi realizada a tabulação dos dados coletados no formulário em uma planilha do *Excel,* a partir da qual foram analisados os voluntários que se enquadraram nos critérios de inclusão do estudo. A partir desse resultado, foram selecionados, por conveniência, os voluntários para comparecimento à avaliação presencial no laboratório de Fonoaudiologia da instituição de ensino onde a pesquisa foi realizada. Essa seleção foi realizada pelos autores a partir dos números atribuídos aos voluntários, aleatoriamente, no momento de inserção de dados na planilha do *Excel*. Entretanto, o número de voluntários selecionados foi não probabilístico e não foi definido ao início da coleta, uma vez que as avaliações aconteceram de acordo com a disponibilidade dos autores para a coleta de dados. Assim, os voluntários foram chamados para a avaliação presencial, que aconteceu dentro do período de, aproximadamente, um ano, e os autores realizaram, em média, uma a duas avaliações por semana. Ao final do período estipulado, as avaliações foram finalizadas com o número de voluntários obtidos.

Para a etapa presencial, foi realizada a avaliação audiológica básica, a aplicação dos testes de processamento auditivo e a avaliação neuropsicológica.

A avaliação audiológica básica foi realizada com objetivo de se avaliar a audição periférica dos voluntários. Foi realizada a meatoscopia, para analisar a integridade do meato acústico externo e membrana timpânica, bilateralmente, avaliando se havia algum impedimento para a realização do exame. Em seguida, foi realizada a Imitanciometria e Audiometria Tonal Liminar (ATL). A Imitanciometria foi realizada com o aparelho AT235h, marca *Interacoustics*, para pesquisa da timpanometria e reflexos estapedianos contralaterais. A timpanometria teve o objetivo de avaliar o funcionamento e integridade da orelha média (OM), bilateralmente, com o tom teste de 226Hz. Foram excluídos os participantes que apresentaram, na timpanometria, curvas do tipo As, Ad, C ou B, de acordo Jerger^(15)^ e Jerger et al.^(16)^, uma vez que esses resultados indicam possível alteração da OM. Foi realizada, também, a Audiometria Tonal Limiar, com o aparelho Piano, da marca *Inventis*, para definir o limiar auditivo nas frequências de 250, 500, 1000, 2000, 3000, 4000, 6000 e 8000 Hz por via aérea e nas frequências de 500, 1000, 2000, 3000 e 4000 Hz por via óssea, quando necessário. A partir da média quadritonal dos limiares das frequências de 500, 1000, 2000 e 4000 Hz, foi realizada a classificação audiométrica de acordo com a Organização Mundial de Saúde^(14)^ e a presença de perda auditiva, de qualquer grau, foi considerada critério de exclusão para o estudo.

Logo após, foi realizada a avaliação comportamental do Processamento Auditivo Central, no qual cada teste foi avaliado seguindo seu padrão de normalidade^(17-21)^. Os resultados foram descritos em categorias binárias de “normal” ou “alterado”.

Na categoria de testes monoaurais de baixa redundância, foi utilizado o teste Fala no Ruído^(17)^, a fim de se avaliar a habilidade de fechamento auditivo. O teste foi realizado a uma intensidade de 40 dBNS (dB nível de sensação) acima da média tritonal, com relação sinal ruído (s/r) ipsilateral de -5 dB. Foram considerados resultados normais aqueles em que houve 70% ou mais de acertos, com diferença entre o resultado com e sem ruído menor que 20%^(17)^.

Na categoria de testes de processamento temporal foram utilizados o *Pitch Pattern Sequence* (PPS)^(18)^ e o *Duration Pattern Sequence* (DPS)^(18)^, para a avaliação da ordenação temporal e o *Random Gap Detection Test* (RGDT)^(19)^ para a avaliação da resolução temporal. Os três foram apresentados a uma intensidade de 50 dB acima da média tritonal. Os testes de ordenação temporal foram realizados sob a forma monótica, com resposta do tipo nomeação. A normalidade foi de acordo com os padrões da versão utilizada^(18)^, sendo 88% de acertos ou mais para o PPS e de 82% ou mais para o DPS, não considerando a inversão como erro. Assim, foram considerados normais os resultados com valor igual ou acima do valor de normalidade mencionado^(18)^. O teste RGDT foi realizado de forma binaural e a normalidade foi definida de acordo com o limiar de detecção encontrado, que deveria ser igual ou menor do que 20 ms, conforme os padrões de Keith^(19)^.

Na categoria de testes de interação binaural foi utilizado o teste *Masking Level Differences* (MLD)^(20)^. O teste foi realizado de forma binaural, com intensidade de 50 dB acima da média tritonal. A normalidade foi de, no mínimo, 10 ms, conforme os padrões de Wilson et al.^(20)^. Dessa forma, os voluntários que tiveram o resultado igual ou maior a 10 ms foram descritos como normais.

Na categoria de testes de escuta dicótica foi utilizado o Teste Dicótico de Dígitos^(21)^, a fim de se avaliar a habilidade auditiva de figura-fundo. O teste foi realizado na tarefa de integração binaural. O critério de normalidade utilizado foi de 95% ou mais acertos^(21)^, assim, os resultados normais deveriam ser iguais ou acima do valor mencionado.

Por fim, foi utilizado o Instrumento de Avaliação Neuropsicológica Breve - NEUPSILIN^(22)^ - que consiste em um teste com diversas tarefas cognitivas de fácil resolução e de rápida aplicação (entre 30 e 40 minutos). Foram realizadas as tarefas que avaliam as seguintes funções: orientação temporoespacial, atenção, memória, habilidades aritméticas, funções executivas (resolução de problemas e fluência verbal). A escolha das tarefas foi realizada com base em achados da literatura, de estudos que pesquisam a relação entre as habilidades supracitadas e suas influências no processamento auditivo^(3,5,9)^. Foi realizada análise quantitativa, considerando-se os escores Z das funções, exceto na função de memória, em que também foi analisado o escore Z da tarefa memória de trabalho. Cada função foi avaliada conforme a pontuação (escore bruto) obtida pelo voluntário, que foi utilizada para calcular o escore Z, considerando o valor médio do grupo no teste e o desvio padrão do seu grupo normativo. Para tal, foram utilizados os grupos normativos de adultos entre 18 e 39 anos e com escolaridade maior do que 9 anos, por serem os grupos correspondentes ao perfil dos voluntários do estudo_._ A partir disso, o desempenho do indivíduo é classificado entre as seguintes categorias: normal, sugestivo de alerta para déficit, sugestivo de déficit, sugestivo de déficit moderado a severo e sugestivo de déficit de gravidade importante^(22)^. Para fins de facilitar a análise dos dados do estudo, as classificações do desempenho foram transformados em categorias binárias, sendo considerados resultados normais aqueles que tiveram o escore Z dentro da categoria “normal” e resultados alterados aqueles que tiveram escore Z dentro das categorias sugestivas de “alerta de déficit”, “déficit”, “déficit moderado a severo” ou “déficit de gravidade importante”.

A avaliação neuropsicológica foi realizada após os testes auditivos. O ambiente de realização da pesquisa era climatizado e foram adotados cuidados a fim de se minimizar o cansaço da aplicação dos testes.

### Análise dos resultados

A análise dos resultados foi ordenada em três partes. Primeiramente, foi realizada uma análise descritiva da amostra. As variáveis qualitativas foram apresentadas em forma de frequência absoluta e relativa, enquanto as variáveis quantitativas passaram pelo teste Shapiro-Wilk. A partir desta análise, os dados quantitativos foram apresentados em forma de mediana e quartis.

Em seguida, para avaliar a relação entre os testes de processamento auditivo e a avaliação neuropsicológica, foi aplicado o teste Qui-Quadrado, no qual o nível de significância das análises foi de 5%. Dessa forma, foi considerado resultado com associação estatisticamente significativa todos aqueles que apresentaram valor *p* inferior a 0,05.

A partir dos resultados da análise gerada pelo teste Qui-Quadrado, foi utilizado o modelo de regressão logística, com objetivo de avaliar a *odds ratio* (razão de probabilidade) das variáveis relacionadas às funções cognitivas sobre os testes de processamento auditivo central. Cada modelo foi composto por todas as variáveis independentes do teste neuropsicológico em relação aos testes de PAC que possuíam valor *p* inferior a 0,2 no teste Qui-Quadrado, ou seja, o modelo de regressão foi gerado a partir de todas as variáveis que apresentaram o valor p<0,2, porém, permaneceram no modelo de regressão apenas as variáveis que alcançaram o valor p<0,05 no modelo matemático, sendo possível avaliar a sua *odds ratio* (razão de probabilidade). Foram incluídas todas as variáveis com valor *p* inferior a 0,2, pois é possível que variáveis com este valor alcancem p<0,05 dentro do modelo de regressão, quando analisadas junto a outras variáveis. Da mesma forma, variáveis que possuem valor p<0,05 isoladamente no Qui-Quadrado podem não apresentar mais tal significância estatística ao serem incluídas com demais variáveis no modelo de regressão. O objetivo da regressão logística utilizada foi avaliar a probabilidade de se apresentar alterações nos testes de PAC a partir dos resultados apresentados nos testes neuropsicológicos.

As análises foram realizadas no *Software* IBM SPSS versão 25.

## RESULTADOS

Após o envio do formulário de solicitação, foram recebidas 1342 respostas de alunos interessados em se voluntariar para o estudo, dos quais 946 se adequavam aos critérios de inclusão. Destes, 101 estudantes participaram da avaliação presencial. Após a aplicação dos critérios de exclusão, foram obtidas 95 avaliações que foram analisadas no estudo. A exclusão dos seis voluntários ocorreu devido à perda auditiva de um participante e presença de curvas timpanométricas do tipo B e C de cinco voluntários. O fluxograma da Figura 1 mostra a relação dos números de participantes em cada etapa.

**Figura 1:** fluxograma das etapas de pesquisa

Dos 95 participantes da pesquisa, 26 (27,7%) eram do sexo masculino e 69 (72,6%) eram do sexo feminino. Em relação à escolaridade, 55 (57,8%) eram estudantes de graduação e 40 (42,1%) eram estudantes da pós-graduação (mestrado e doutorado). Quanto à modalidade da escola em que fizeram o ensino médio, 60 voluntários (63,2%) eram provenientes de escola pública, enquanto 35 voluntários (36,8%) eram provenientes de escola particular. A idade mediana da amostra foi de 25 anos, sendo que o primeiro quartil foi de 23 anos e o terceiro quartil foi de 30 anos.

Quanto aos testes de processamento auditivo e NEUPSILIN, os resultados foram descritos em variáveis categóricas binárias, descritas como “normal” ou “alterado”. Assim, nos testes de processamento auditivo, foram considerados alterados os resultados fora do padrão de referência de cada teste. Da mesma forma, foram considerados alterados, no teste neuropsicológico, quaisquer resultados diferentes de “normal” após análise dos escores Z do NEUPSILIN, incluindo aqueles descritos como sugestivo de “alerta de déficit”, “déficit”, “déficit moderado a severo” ou “déficit de gravidade importante”. Nas Tabelas 1 e 2 estão apresentados os resultados das avaliações.

**Tabela 1:** Resultados dos testes de PAC (n = 95)

**Variáveis**		**n**	**%**
**Fala no Ruído**	Normal	64	67,4
	Alterado	31	32,6
**MLD**	Normal	74	77.9
	Alterado	21	22,1
**PPS**	Normal	60	63,2
	Alterado	35	36,8
**DPS**	Normal	77	81,1
	Alterado	18	18,9
**RGDT**	Normal	82	86,3
	Alterado	13	13,7
**TDD**	Normal	86	90,5
	Alterado	9	9,5

**Legenda:** MLD = Masking Level Difference; PPS = Pitch Pattern Sequence; DPS = Duration Pattern Sequence; RGDT = Random Gap Detection; TDD = Teste Dicótico de Dígitos Test; PAC = Processamento Auditivo Central; Normal = resultados considerados normais de acordo com o padrão de referência de cada teste; Alterado = resultados considerados alterados de acordo com o padrão de referência de cada teste

**Tabela 2:** Resultados do Neupsilin (n = 95)

**Variáveis**		**n**	**%**
**Orientação temporoespacial**	Normal	86	90,5
	Alterado	9	9,5
**Atenção**	Normal	93	97,9
	Alterado	2	2,1
**Memória**	Normal	84	88,4
	Alterado	11	11,6
**Memória de trabalho**	Normal	86	90,5
	Alterado	9	9,5
**Habilidades aritméticas**	Normal	76	80,0
	Alterado	19	20,0
**Linguagem**	Normal	85	89,5
	Alterado	10	10,5
**Funções executivas (resolução de problemas)**	Normal	85	89,5
	Alterado	10	10,5
**Funções executivas (fluência verbal)**	Normal	84	88,4
	Alterado	11	11,6

**Legenda:** Normal = resultados dentro da classificação “normal”, de acordo com o padrão de referência do teste; Alterado = resultados dentro das categorias sugestivas de “alerta de déficit”, “déficit”, “déficit moderado a severo” ou “déficit de gravidade importante”, de acordo com o padrão de referência do teste

Foi realizada a análise de associação entre as variáveis categóricas binárias dos testes de processamento auditivo e testes neuropsicológicos, entretanto, não houve associações estatisticamente significativas entre as funções cognitivas avaliadas e os testes Fala no Ruído, PPS, DPS e MLD. Houve associação estatisticamente significativa entre os testes RGDT e TDD com algumas variáveis neuropsicológicas estudadas.

Na análise de associação entre o RGDT e a avaliação neuropsicológica, houve duas variáveis com resultado estatisticamente significante (*p* valor <0,05), sendo essas as tarefas de atenção (*p* valor = 0,017) e de fluência verbal (*p* valor = 0,041). É importante salientar, em relação à atenção, que todos os casos (100%) que apresentaram resultado normal no RGDT também tiveram resultado normal na tarefa de atenção. Na Tabela 3, estão demonstrados os resultados da associação entre o RGDT e avaliação neuropsicológica.

**Tabela 3:** Resultado da associação entre RGDT e a avaliação neuropsicológica

**Variáveis**	**Teste RGDT**		
	**Normal**	**Alterado**	**valor p** ^*^
**Orientação temporoespacial**			
Normal	73 (89%)	13 (100%)	0,353
Alterado	9 (11%)	0 (0%)	
**Atenção**			
Normal	82 (100%)	11 (84,6%)	**0,017**
Alterado	0 (0%)	2 (15,4%)	
**Memória**			
Normal	74 (91,5%)	10 (76,9%)	0,172
Alterado	8 (9,8%)	3 (23,1%)	
**Memória de trabalho**			
Normal	75 (91,5%)	11 (84,6%)	0,606
Alterado	7 (8,5%)	2 (15,4%)	
**Habilidades aritméticas**			
Normal	66 (80,5%)	10 (76,9%)	0,719
Alterado	16 (19,5%)	3 (23,1%)	
**Linguagem**			
Normal	73 (89%)	12 (92,3%)	0,999
Alterado	9 (11%)	1 (7,7%)	
**Resolução de problemas**			
Normal	75 (91,5%)	10 (76,9%)	0,136
Alterado	7 (8,5%)	3 (23,1%)	
**Fluência verbal**			
Normal	75 (91,5%)	9 (69,2%	**0,041**
Alterado	7 (8,5%)	4 (30,8%)	

* Teste Qui Quadrado

Legenda: RGDT= Random Gap Detection Test

Além do RGDT, o Teste Dicótico de Dígitos também apresentou associação estatisticamente significativa com uma das variáveis do NEUPSILIN, sendo essa a memória de trabalho (*p* valor = 0,038), como demonstrado na Tabela 4.

**Tabela 4:** Resultado da associação entre Teste Dicótico de Dígitos e a avaliação neuropsicológica

**Variáveis**	**Teste Dicótico de Dígitos**		
	**Normal**	**Alterado**	**valor p** ^*^
**Orientação temporoespacial**			
Normal	77 (89,5%)	9 (100%)	0,594
Alterado	9 (10,5%)	0 (0%)	
**Atenção**			
Normal	85 (98,8%)	8 (88,9%)	0,181
Alterado	1 (1,2%)	1 (11,1%)	
**Memória**			
Normal	77 (89,5%)	7 (77,8%)	0,279
Alterado	9 (10,5%)	2 (22,2%)	
**Memória de trabalho**			
Normal	80 (93%)	6 (66,7%)	**0,038**
Alterado	6 (7%)	3 (33,3%)	
**Habilidades aritméticas**			
Normal	70 (81,4%)	6 (66,7%)	0,377
Alterado	16 (18,6%)	3 (33,3%)	
**Linguagem**			
Normal	78 (90,7%)	7 (77,8%)	0,240
Alterado	8 (9,3%)	2 (22,2%)	
**Resolução de problemas**			
Normal	79 (91,9%)	6 (66,7%)	0,051
Alterado	7 (8,1%)	3 (33,3%)	
**Fluência verbal**			
Normal	76 (88,4%)	8 (88,9%)	0,999
Alterado	10 (11,6%)	1 (11,1%)	

* Teste Qui Quadrado

Em seguida, foi aplicado o modelo de regressão logística para todas as associações dos testes de PAC com as variáveis neuropsicológicas que obtiveram valor p<0,2, a fim de investigar a relação entre tais variáveis. A partir disso, o modelo logístico avaliou quais alcançaram valor p<0,05 e apresentou a *odds ratio* (razão de probabilidade) entre elas, ou seja, a probabilidade dos que apresentaram alteração nos testes neuropsicológicos apresentarem alterações, também, nos testes de PAC, em relação aos que não tiveram as respectivas variáveis neuropsicológicas alteradas. Nenhuma variável neuropsicológica analisada com os testes de Fala no Ruído, PPS, DPS e MLD alcançaram o valor p<0,05 no modelo de regressão logística e, por isso, não há modelo a ser apresentado para tais testes.

Apesar da atenção ter apresentado, isoladamente, associação estatisticamente significativa com o RGDT no teste Qui-Quadrado, ao ser aplicado o modelo de regressão logística com as variáveis que haviam apresentado valor p<0,2 em relação ao RGDT, apenas a fluência verbal permaneceu no modelo matemático com valor p<0,05, sendo possível avaliar a sua *odds ratio* (razão de probabilidade). Assim, foi demonstrado que a alteração na fluência verbal aumenta em 4,7 vezes a chance da alteração no RGDT, em relação à não alteração da fluência verbal. A capacidade de classificação do modelo foi de 86,3%, ou seja, quase a totalidade das estimativas geradas pela regressão são coincidentes com o resultado real da variável.

Além disso, um segundo modelo de regressão, desta vez, em relação ao TDD, obteve variáveis com valor p<0,05 ao final, sendo possível avaliar a *odds ratio* (razão de probabilidade). O modelo demonstrou que a alteração na memória de trabalho e na resolução de problemas aumentam em quase 8 vezes e em 6,6 vezes, respectivamente, a chance de se ter um resultado alterado no teste de escuta dicótica (TDD), em relação à não alteração em tais funções cognitivas. Tais resultados estão expostos na Tabela 5.

**Tabela 5:** Modelo de regressão logístico

	**OR**	**Wald**	**df**	**valor p**	**95% C.I. para OR**		**Tabela de Classificação**
**Variável resposta: Teste RGDT**							
Constante	0,120	36,125	1	0,000			
Fluência verbal	4,762	4,708	1	0,030	1,163	19,498	86,3%
**Variável resposta: Teste Dicótico de Dígitos**							
Constante	0,051	32,888	1	0,000			
Memória de trabalho	7,814	5,469	1	0,019	1,395	43,770	91,6%
Resolução de problemas	6,652	4,762	1	0,029	1,213	36,479	

**Legenda:** OR = razão de probabilidade; Tabela de classificação: capacidade explicativa do modelo

As tabelas com os resultados da análise de associação dos demais testes de processamento auditivo - Fala no Ruído, PPS, DPS e MLD - com as funções cognitivas avaliadas estão disponíveis como Material Suplementar. Encorajamos a todos os leitores a consultá-las para verificar os resultados de cada teste.

## DISCUSSÃO

O presente estudo teve como objetivo analisar a influência das funções cognitivas nos resultados dos testes de Processamento Auditivo Central na população de estudantes universitários. Tal temática é importante devido ao fato de que diferentes processos cognitivos podem influenciar no momento da avaliação do processamento auditivo, sendo necessário que esses processos sejam conhecidos a fim de se propiciar uma avaliação mais precisa do indivíduo avaliado^(1,3)^.

Neste estudo, a população foi composta por estudantes universitários a nível de graduação e pós graduação, sendo que a maior parte dos voluntários foram mulheres, estudantes de graduação, provenientes de escola pública. Na literatura, podem ser encontrados diversos estudos a respeito de aspectos cognitivos e do PAC em crianças^(2,9,10),^ entretanto, estudantes acadêmicos universitários ainda não são o foco das pesquisas, embora a população também seja alvo de queixas acadêmicas e de auditivas^(4,13)^.

A literatura demonstra uma variabilidade de funções cognitivas que podem influenciar na avaliação do processamento auditivo^(3)^. A princípio, os resultados do estudo encontraram associação estatisticamente significativa entre a resolução temporal e as tarefas de atenção e fluência verbal, porém, a partir do modelo de regressão logística, apenas a fluência verbal permaneceu como uma variável que apresenta relação com a alteração no teste RGDT.

Uma revisão sistemática da literatura realizada em 2023 demonstrou que a ordenação temporal esteve mais relacionada, nos estudos incluídos, com os processos cognitivos de memória de trabalho, testes de inteligência e medidas globais cognitivas, apesar de também apontarem estudos que não obtiveram essa associação. A maior parte dos trabalhos encontrados na literatura que estudaram a relação entre o processamento temporal e funções cognitivas utilizou testes de ordenação temporal para avaliação^(3,23,24)^. Demais estudos que buscaram estudar a resolução temporal, em sua maioria, não encontraram associações estatisticamente significativas entre esta habilidade e diferentes domínios cognitivos, como atenção, memória e inteligência^(3, 24)^.

Neste sentido, o presente estudo foi de encontro à literatura, pois não foram encontradas associações entre as funções cognitivas estudadas e a ordenação temporal, avaliada pelos testes PPS e DPS. Apesar disso, a maior parte dos participantes que apresentaram resultado normal nestes testes também apresentaram resultado normal para as tarefas de atenção e memória de trabalho. Houve associação estatisticamente significativa entre a resolução temporal, avaliada pelo teste RGDT, e as tarefas de atenção e de fluência verbal. O resultado contrastante com a literatura pode ser explicado pelo fato exposto anteriormente, de que a maioria das pesquisas não objetiva estudar a resolução temporal, além da amostra reduzida de participantes de tais estudos^(3,24)^.

Apesar de não ter permanecido no modelo de regressão logístico junto à fluência verbal, a associação estatisticamente significativa encontrada entre o RGDT e a atenção no teste Qui-Quadrado nos indica que essa pode ser uma função cognitiva importante para a realização da tarefa de resolução temporal. O RGDT consiste em um teste no qual o sujeito deve identificar, dentre uma série de estímulos, se ele ouviu a presença de um ou dois tons^(19)^. Embora o tempo de teste não seja longo, é plausível o resultado encontrado de que a atenção atua como um papel crucial na sua realização, sendo sugestivo de que esta seja uma função necessária para que haja consistência nas respostas durante a avaliação. Assim, a atenção aparenta ser um fator protetivo, associada positivamente ao desempenho adequado no RGDT e pode ser considerada uma função importante para a realização deste teste.

Além da atenção, a fluência verbal também apresentou resultado estatisticamente significativo e se manteve no modelo de regressão como uma variável com forte influência no RGDT. De acordo com os resultados encontrados, há maior tendência de se apresentar alterações no RGDT (cerca de 4,7 vezes de chance a mais) quando há alteração no teste de fluência verbal. No instrumento NEUPSILIN, a fluência verbal é apontada como um teste de funções executivas. As FE incluem uma série de processos cognitivos, como memória de trabalho, controle inibitório e flexibilidade cognitiva^(6)^, que são importantes para o gerenciamento de tarefas, planejamento e monitoramento de outras funções cognitivas, como a de atenção sustentada. A tarefa de fluência verbal é um dos principais indicadores utilizados para avaliar funções executivas, uma vez que envolve a capacidade de organização do pensamento e velocidade de produção lexical e acesso ao léxico^(25)^, o que sugere que os processos cognitivos de planejamento e velocidade de pensamento estejam envolvidos na realização de ambos os testes e sejam funções importantes na realização do RGDT. Portanto, é possível que a dificuldade no planejamento cognitivo e velocidade de pensamento aumentem as chances de se apresentar alteração em tarefas de resolução temporal. Entretanto, é importante que este resultado seja avaliado com cautela, uma vez que não é um achado frequente na literatura.

Dentre as funções cognitivas, a memória de trabalho é um dos fatores mais estudados em relação à sua influência no desempenho dos testes de processamento auditivo^(3)^. Neste estudo, a memória de trabalho e a resolução de problemas demonstraram influência, especificamente, para a realização do Teste Dicótico de Dígitos. A escuta dicótica se refere à capacidade de separar ou integrar informações de diferentes estímulos auditivos apresentados, ao mesmo tempo, nas duas orelhas^(1)^. De acordo com os resultados apresentados no modelo de regressão, a alteração na memória de trabalho aumenta em 8 vezes as chances de se apresentar alteração no TDD do que quando não há dificuldades na memória de trabalho. Outros estudos já apontavam a importância dessa função para escuta dicótica, utilizando diferentes testes, seja na tarefa de integração ou de separação binaural^(9,26)^. Durante a realização dos testes de escuta dicótica, é essencial que o avaliador tenha conhecimento de que a memória de trabalho é recrutada em resposta a estímulos dicóticos, o que pode influenciar no desempenho do indivíduo avaliado. Vale ressaltar que, apesar do NEUPSILIN avaliar a memória de trabalho separadamente das funções executivas, ela é um dos seus principais componentes, pelo fato de possibilitar a manipulação de informações^(6)^, incluindo auditivas, como é necessário que seja realizado durante a execução das tarefas de escuta dicótica^(9,26)^.

Além da memória de trabalho, o modelo de regressão indicou que a alteração na tarefa de resolução de problemas aumenta em 6,6 vezes a chance de alteração no TDD. Esse achado nos sugere que também há o envolvimento de processos cognitivos de planejamento, priorização, estratégia e flexibilidade cognitiva na resposta aos estímulos dicóticos. Assim, reitera-se a necessidade de se considerar as funções executivas no momento da avaliação da escuta dicótica, especialmente, se a avaliação estiver sendo realizada para o diagnóstico diferencial do TPAC e de outros transtornos, como o TDAH, em que as dificuldades de funções executivas são comuns^(3,9,11,27,28)^.

A literatura também indica que há relação entre o fechamento auditivo com algumas habilidades cognitivas, especialmente as funções executivas de controle inibitório e memória de trabalho^(3,7,12)^. Em nosso estudo, não encontramos associação estatisticamente significativa entre essas variáveis. Entretanto, esse resultado pode ser explicado em razão de que as pesquisas que encontraram tais associações utilizaram testes de fala monoaural de baixa redundância estruturados com sentenças e com sinais auditivos complexos ou degradados^(3,7,12)^. O teste Fala no Ruído consiste na repetição de monossílabos apresentados ipsilateralmente com ruído branco. Dessa forma, a não associação do teste Fala no Ruído com as funções cognitivas avaliadas nos sugere que a repetição imediata de monossílabos, ainda que no ruído, não recruta as funções de memória de trabalho e controle inibitório da mesma forma que os outros testes da mesma categoria.

É importante ressaltar que o estudo apresenta a fragilidade de ter utilizado uma amostra que não passou por cálculo amostral, a qual foi composta de 95 estudantes da graduação e pós-graduação, recrutados por conveniência, com uma grande porcentagem de resultados normais. Assim, é necessário que, futuramente, sejam realizados estudos com cálculo amostral, a fim de que seja possível avaliar de forma representativa a população estudada. Sugere-se, também, que sejam utilizados testes neuropsicológicos mais específicos para as funções cognitivas que se mostraram mais relevantes, neste estudo, durante a avaliação do processamento auditivo, principalmente a atenção e as funções executivas em suas subcategorias. Além disso, sugere-se a realização de testes eletrofisiológicos que apresentam associação com processos cognitivos, como os potenciais corticais, para complementar a avaliação^(3,29)^.

Apesar das fragilidades apresentadas, reconhece-se a importância deste estudo, uma vez que a população avaliada não é o foco da maioria das pesquisas encontradas atualmente na literatura. Ademais, ainda não há um consenso a respeito da influência das funções cognitivas nos testes de processamento auditivo^(3)^. Embora não tenham sido encontradas associações estatisticamente significativas entre algumas funções cognitivas e o desempenho em alguns testes de PAC, entende-se, por esse resultado, que os testes utilizados para avaliar o processamento auditivo estão cumprindo seu propósito e apresentam confiabilidade. Frascá e colegas^(30)^ já haviam demonstrado, por meio de um estudo de teste e reteste, que as avaliações apresentam um bom nível de confiabilidade, e o presente estudo reforça esses achados.

Ainda assim, é imprescindível que os aspectos cognitivos e neuropsicológicos sejam considerados na avaliação do processamento auditivo, a fim de que haja uma análise mais precisa do indivíduo^(1,5)^. De acordo com os resultados encontrados neste estudo, a atenção, memória de trabalho, planejamento cognitivo, velocidade de processamento e flexibilidade cognitiva apresentaram relação com os testes de PAC avaliados, sugerindo que a avaliação comportamental do processamento auditivo pode recrutar tais funções cognitivas. Portanto, a avaliação multidisciplinar, com o fonoaudiólogo, neuropsicólogo, entre outros profissionais, é de extrema importância, especialmente para esclarecer os resultados dentro do contexto de um diagnóstico diferencial entre transtornos.

## CONCLUSÃO

A literatura indica que diferentes funções cognitivas podem influenciar nos testes de PAC em adultos. Os resultados do presente estudo sugeriram que a atenção, memória de trabalho, planejamento cognitivo, velocidade de processamento e flexibilidade cognitiva são recrutados durante a avaliação de PAC, sendo que a alteração na fluência verbal aumentou as chances de alteração no teste de resolução temporal e a alteração na memória de trabalho e na resolução de problemas aumentaram as chances de alteração no teste de escuta dicótica. Além disso, a atenção parece ser um fator protetivo para um bom desempenho na realização do teste de resolução temporal. Assim, é essencial considerar o desempenho de tais funções durante a avaliação do processamento auditivo central para um diagnóstico mais preciso do TPAC na população adulta e universitária. Novas pesquisas podem ampliar esta reflexão sobre a análise interpretativa dos testes e o diagnóstico de TPAC na população estudada.

## Supplementary Material

Supplementary material accompanies this paper.

Supplementary Table 1 Results of the association between the Speech-in-Noise test and neuropsychological assessment

Supplementary Table 2 Results of the association between MLD and neuropsychological assessment

Supplementary Table 3 Result of the association between PPS and neuropsychological assessment

Supplementary Table 4 Result of the association between DPS and neuropsychological assessment

This material is available as part of the online article from https://doi.org/10.1590/2317-1782/e20250036en
